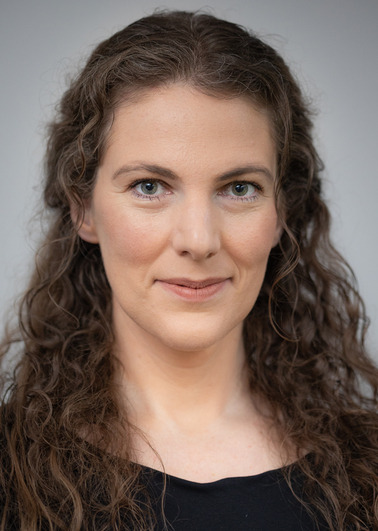# Advances in the Sensing and Treatment of Wound Biofilms

**DOI:** 10.1002/anie.202112218

**Published:** 2022-02-03

**Authors:** Sorour Darvishi, Shima Tavakoli, Mahshid Kharaziha, Hubert H. Girault, Clemens F. Kaminski, Ioanna Mela

**Affiliations:** ^1^ Department of Chemical Engineering and Biotechnology University of Cambridge Philippa Fawcett Drive Cambridge CB3 0AS UK; ^2^ Department of Chemistry and Chemical Engineering École Polytechnique Fédérale de Lausanne 1951 Sion Switzerland; ^3^ Department of Chemistry-Ångstrom Laboratory Uppsala University SE75121 Uppsala Sweden; ^4^ Department of Materials Engineering Isfahan University of Technology Isfahan 84156-83111 Iran

**Keywords:** biofilms, biosensors, medicinal chemistry, nanotechnology, wound biofilms

## Abstract

Wound biofilms represent a particularly challenging problem in modern medicine. They are increasingly antibiotic resistant and can prevent the healing of chronic wounds. However, current treatment and diagnostic options are hampered by the complexity of the biofilm environment. In this review, we present new chemical avenues in biofilm sensors and new materials to treat wound biofilms, offering promise for better detection, chemical specificity, and biocompatibility. We briefly discuss existing methods for biofilm detection and focus on novel, sensor‐based approaches that show promise for early, accurate detection of biofilm formation on wound sites and that can be translated to point‐of‐care settings. We then discuss technologies inspired by new materials for efficient biofilm eradication. We focus on ultrasound‐induced microbubbles and nanomaterials that can both penetrate the biofilm and simultaneously carry active antimicrobials and discuss the benefits of those approaches in comparison to conventional methods.

## Graphical abstract

Wound biofilms are a challenging problem for modern medicine. Advances in the areas of sensors and reporters that can accurately detect early biofilm formation in the wound, and new materials to treat wound biofilms, offer promise for better detection and treatment. This review focuses on novel approaches to biofilm eradication and detection and compares them to conventional methods.

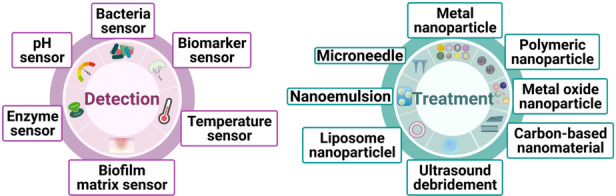

## 1 Introduction

Antibiotic‐resistant infections are threatening to become one of the major health crises in the world, with enormous socio‐economic consequences. Pathogenic bacteria are gaining resistance to modern, last‐resort antibiotics at an alarming rate. In this context, microbial biofilms represent a particularly challenging problem. Biofilm‐associated infections lead to a severe economic loss^[1]^ and over a half million deaths annually. The cost of treating biofilms is estimated at around USD 94 billion annually.^[2]^ In 2007, the Center for Disease Control reported that around 1.7 million hospital‐acquired infections were due to biofilms.^[3]^ Biofilms are assemblages of one or more types of microorganisms that can grow on many different surfaces, ranging from packaging materials to soft tissues. Bacteria in biofilms show distinctive differences to their planktonic counterparts, namely the production of extracellular polymeric substances (EPS), up‐ or down‐regulation of genes, and slower growth rates.^[4]^ A biofilm, in nature, is typically made up of several microbial species attached to EPS that surrounds and protects cells. A typical EPS matrix contains polysaccharides, proteins, lipids, and extracellular DNA (eDNA).^[5]^ Moreover, biofilms resist host immune responses and are much less susceptible to antibiotics. They are involved in numerous subacute and chronic infections and can cause persistent infections through microbial accumulation in the EPS of the biofilm.^[6]^

One of the acute problems associated with biofilms is their formation on wound surfaces. Bacteria in this form impede the healing of 60 % of chronic wounds and 6 % of acute wounds.^[7]^ Anti‐biofilm wound dressings represent a significant part of the market, with USD 570.1 million in 2019 and a compound annual growth rate (CAGR) of 9.1 % from 2020 to 2027, reflecting its importance. Additionally, increasing antibiotic resistance and chronic wound infection rates are expected to increase demand for early diagnosis and treatment of chronic wound biofilms.

This review focuses on recent advances and current state‐of‐the‐art in the field of wound biofilm detection and eradication. It focuses on novel, sensor‐based approaches that show promise for early, accurate detection of biofilm formation on wound sites and that can be translated from laboratory applications to point‐of‐care settings. Then, we discuss technologies inspired by new materials that show potential for efficient biofilm eradication. The ultrasound‐induced microbubbles and nanomaterials have the potential to both penetrate the biofilm and simultaneously carry active antimicrobials. Numerous detailed reviews describe the biology of biofilms and methods for their characterization and eradication thoroughly. However, only a limited number of reviews focus on detecting and eradicating biofilms that form on wound sites. The focus of these reviews has been on small subsets of existing methods for eradication and diagnosis.^[8]^ Our review focuses specifically on wound biofilms and their early detection through new technologies that can be used in point‐of‐care applications, and simultaneously, on new technologies that can be used for efficient eradication of wound biofilms with the minimal patient inconvenience. With this review, we hope to draw the attention of scientists with expertise in sensor technology, drug delivery, and microbiology to the pressing issue of wound biofilms and, through discussing exciting new technologies and chemical avenues, to stimulate further work in these areas.

## 2 Chronic Wound Biofilms

All open wounds contain microorganisms from endogenous or exogenous sources because the innate protective covering of the skin is compromised.^[9]^ A wound biofilm is formed when certain microorganisms, mostly pathogenic bacteria, adhere to the wound surface. Biofilms are formed by a three‐dimensional (3D) matrix that provides protection and cohesion for bacteria growing in wound sites.^[10]^ The host's immune system clears microbes under normal physiological conditions, which leads to a normal wound healing process. Microbes are more likely to attach to wound surfaces when the immune system is dysfunctional or dysregulated. With the secretion of EPS, a biofilm is formed.^[10a]^ Macrophages and neutrophils are key players in protecting the body's innate immune system from pathogens;^[11]^ they are vital components of a highly effective defense mechanism against planktonic bacteria. However, bacteria trapped within biofilm structures are effectively protected from phagocytic attack by neutrophils and macrophages.^[12]^ A biofilm also produces leukocyte‐inactivating substances, resulting in a condition known as “frustrated phagocytosis”.^[13]^

**Figure 1:**
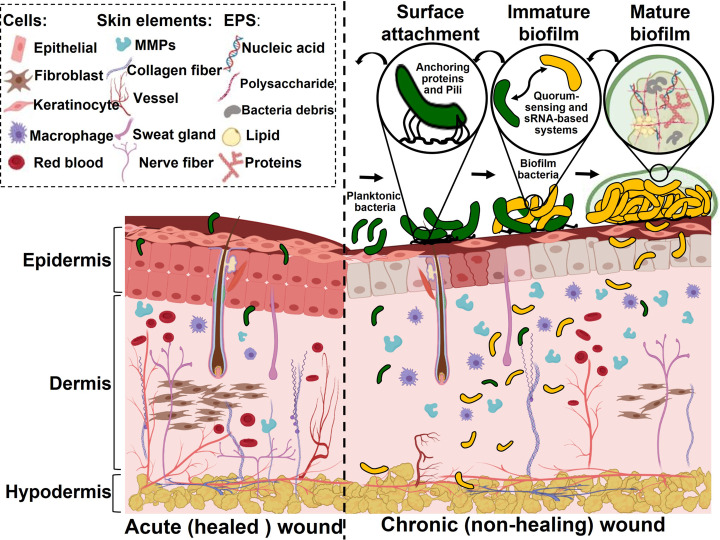
Differences between the healing of an acute wound and a chronic wound: A biofilm is formed in the chronic wound. The biofilm formation stages are represented: Specific anchoring proteins and pili allow planktonic bacteria to attach to wound surfaces. A quorum‐sensing system and sRNA‐based systems transform planktonic bacteria into biofilm bacteria after successful attachment. Microcolonies gradually transform into mature biofilms.

Not all bacterial species found in wounds cause infection.^[14]^ Detecting wound infections can be challenging, especially in wounds with pathogenic biofilms. Therefore, an approach to detect specific bacterial species on the wound site is vital. Different in vitro and in vivo studies have investigated biofilms on wounds. Biofilms can be divided into two main categories: commensal and pathogenic. Pathogenic biofilms differ from commensal ones in several ways, including the presence of more upregulated genes. This promotes excessive levels of degradation enzymes (matrix metalloproteinases (MMPs)), the growth of EPS, and increased microbial proliferation and dissemination (which characterize chronic wounds).[^10a^, ^15^] The establishment of pathogenic biofilms leads to an upregulation of the immune responses, leading to chronic inflammation. Figure 1 provides a comparison between an acute healing wound and a chronic nonhealing wound.

The host's immune system controls or destroys pathogenic bacteria early in the development of chronic wounds. However, a biofilm matrix can develop when bacteria successfully attach to the wound surface.^[16]^ The biofilm phenotype of bacteria is induced by complex intracellular signals that alter planktonic bacteria gene expression profiles. By this time, bacteria have formed microcolonies which become biofilms as they work in conjunction with quorum‐sensing systems and bacterial small RNA‐based systems. A bacterial small RNA (sRNA) is a regulatory RNA that is approximately 40–500 nucleotides long.^[17]^ There are two general mechanisms by which sRNAs form biofilms. The first involves sRNAs interacting with other RNAs, and the second involves proteins binding to sRNAs. By altering the accessibility of ribosome binding sites (RBS) or enhancing degradation by ribonuclease, base‐pairing between sRNAs and their targeted mRNAs can alter mRNA translation and stability and thus influence gene expression.^[18]^

The microcolonies gradually mature into a matrix of EPS and inflammatory materials from the wound in a cellular matrix surrounded by normal skin cells. The biofilm volume is dominated by EPS, which accounts for 90 %, while bacteria account for 10 %.^[19]^ Biofilms eventually form stalk‐like structures. Then, the structure is dispersed and detached, forming new colonies or incorporating existing biofilms in a chronic wound.^[8c]^ Wound biofilm formation is a dynamic process, and it has been found that bacteria can form a mature biofilm on a wound bed within 24 h.^[20]^ When the biofilm is well established, it will not be destroyed by the host immune system spontaneously. However, the formed biofilm can delay wound healing, require tissue amputation, and ultimately lead to sepsis and death.^[2]^ Studies have shown that chronic wounds contain a pathogenic biofilm more than 60 % of the time, whereas only 6 % of acute wounds contain biofilms.^[7a]^

A chronic wound biofilm comprises multiple bacteria groups, generally with different genotypes, and which are further held together by EPS.^[21]^ For instance, chronic venous leg ulcers contain *S. aureus* (93.5 % of the investigated ulcers), *Enterococcus faecalis* (71.7 %), *P. aeruginosa* (52.2 %), coagulase‐negative *Staphylococci* (45.7 %), *Proteus* species (41.3 %), and anaerobic bacteria (39.1 %).^[22]^ Table 1 shows the participation of various bacterial groups presenting in chronic wound biofilms that cause associated diseases for the host body. In most studies, *S. aureus* is present in chronic wounds as the most prevalent biofilm bacteria.^[23]^ A significant portion of chronic wounds is colonized with *P. aeruginosa* in deep dermal tissues, and a wound infected with *P. aeruginosa* causes significant wound area increases compared to a wound not infected by the bacterium.^[24]^ It is possible to delay or even prevent wound healing by introducing *P. aeruginosa* into the wound bed.^[25]^ Consequently, preventing biofilm formation and then detecting a formed biofilm at the wound, especially at an early stage of formation, is vitally important.

**Table 1:** The most common bacteria present in chronic wounds biofilm and associated diseases for the host body.

Organism	Gram‐negative/positive	Biofilm‐associated diseases	Ref.
*Staphylococcus aureus*	Positive	Skin and soft tissue infections, abscesses (boils), osteomyelitis, indwelling medical device infection, chronic rhinosinusitis	[26]
*Enterococcus faecalis*	Positive	Chronic wound infection, urinary tract infections, caries, endocarditis, and bacteremia	[27]
*Pseudomonas aeruginosa*	Negative	Chronic wound infection, especially in burn wounds, respiratory system infections, urinary tract infections, dermatitis, soft tissue infections, bacteremia, bone and joint infections, gastrointestinal infections	[28]
Coagulase‐negative *Staphylococci*	Negative	Urinary tract infection, breast abscess, skin and soft tissue infection such as cellulitis, furunculosis, and native valve endocarditis	[29]
*Proteus* species	Negative	Wound infections, burn infections, respiratory tract infections, and bacteremia	[30]

## 3 Wound Biofilm Detection

**Figure 2:**
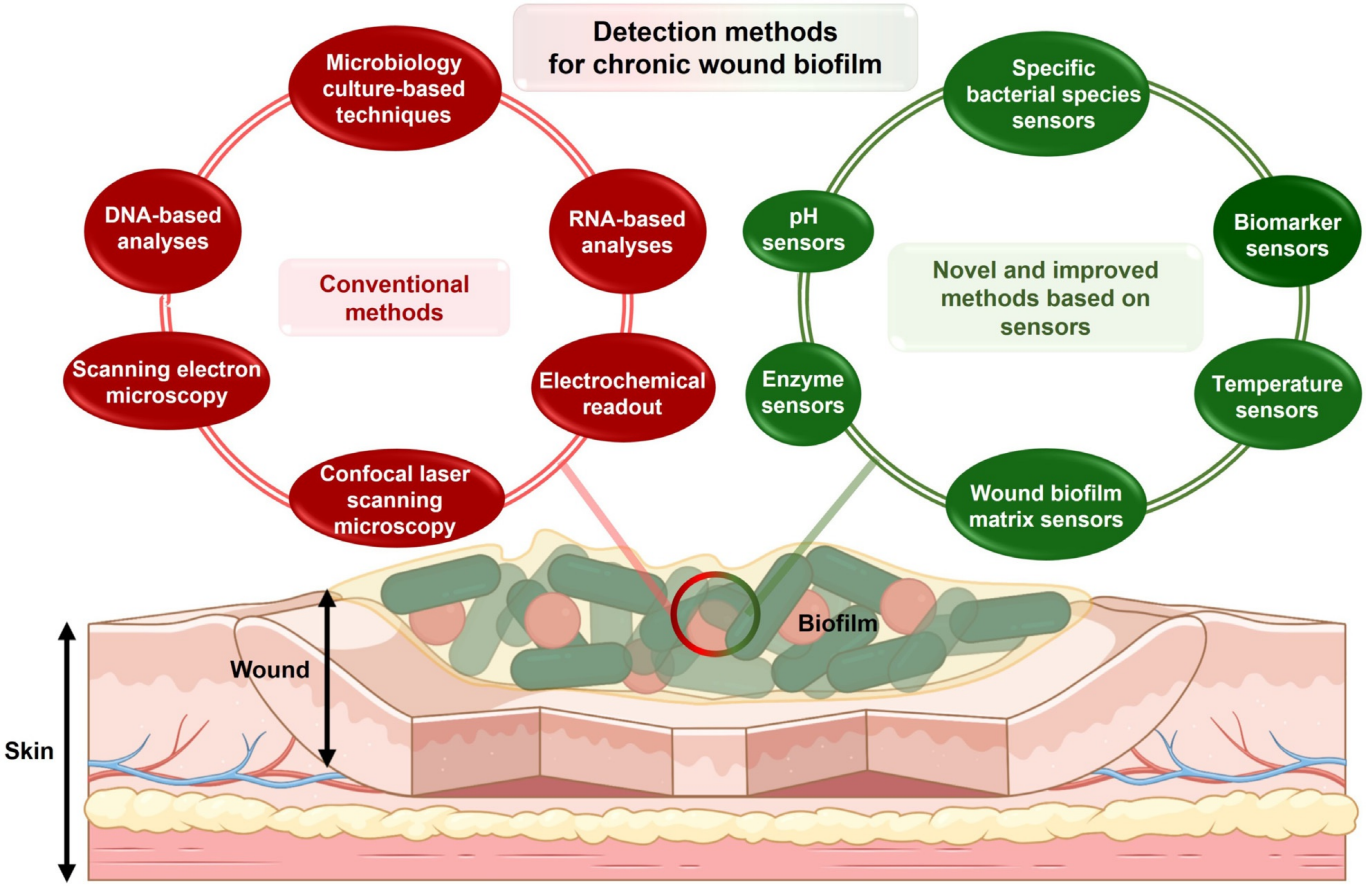
Methods for detection of chronic wound biofilm. Methods are separated into conventional and novel methods. Novel methods discussed in the review paper are based on sensor‐based readouts.

Although some clinical symptoms of the formation of pathogenic biofilms, such as yellow exudate, pale wound bed, necrotic tissue, and clear tissue fluid, are distinguishable, the bacterial aggregates in wound biofilms are not discernable by the unaided eye as they usually measure less than 100 μm in size.^[31]^ Therefore, different methods have been developed to detect microorganisms on the wound site (Figure 2). Traditionally, biofilm detection techniques are categorized into microbiology assays, molecular assays, and imaging assays. While not the focus of this review, these methods are briefly described below:

Microbiology culture‐based techniques have been used to detect viable culturable bacteria in the wound. However, diagnosis of chronic wound infection using this method lacks accuracy and was demonstrated to considerably underestimate the existence of bacteria in the wound.[^31^, ^32^] The biofilm is sampled directly from the wound site through a surgical procedure or sonication.^[33]^ Inaccuracies in the detection may arise from the fact that many bacteria do not form colonies in normal culturing conditions (slow‐growing variants, dormant persister cells, and anaerobic bacteria).^[34]^ For these reasons, some studies have been misconstrued and have underestimated bacterial populations in wounds.^[35]^

DNA‐ and RNA‐based analyses of biofilm bacterial species are more precise than culturing methods and can detect unculturable cells and samples with mixed species (anaerobic and aerobic bacteria).^[36]^ Molecular sequencing methods such as denaturing gradient gel electrophoresis (DGGE) have also been used in biofilm detection. As part of the investigation of chronic wound biofilms, this method was used in conjunction with 16S rRNA‐PCR to characterize biofilms that are complex and multispecies in nature.^[37]^ Other sequencing methods for the detection of chronic wound biofilms include partial ribosomal amplification and pyrosequencing (PRAPS), whole ribosomal amplification, cloning Sanger sequencing (FRACS), partisan ribosomal amplification, denaturing gradient gel electrophoresis Sanger sequencing (PRADS), and PNA‐FISH.[^22^, ^38^]

Nevertheless, some limitations are associated with DNA‐based technologies. The three primary concerns are the possibility of DNA contamination from the clinical environment, no cell viability information, and not distinguishing between biofilms and planktonic microbes. Combined DNA‐based and messenger RNA analyses, however, may be able to recognize the organism's genotype.^[33]^ Some bacterial species, such as mycobacterium, cannot be detected by 16S rRNA sequencing.^[39]^ Moreover, sequencing results may correspond to microorganisms not documented in existing databases, rendering the technique powerless to identify them.^[40]^ Molecular assays, in general, are accessible and cost‐effective. By using these methods, wound biofilms can be analyzed, and the bacterial populations can be detected and quantified rapidly.^[41]^

Wound biofilms can also be detected using microscopy. To identify biofilms in wounds, confocal laser scanning microscopy (CLSM) and scanning electron microscopy (SEM), which examine biofilms by imaging their polysaccharide matrix and bacterial morphologies, have also been used.^[42]^ Through the use of a microscope, interconnected fibrous networks of EPS are revealed as well as crosslinking between bacterial cells within the polymeric matrix, enzyme activity, species, and viability of microcolonies.[^42^, ^43^] Electrochemical bioimaging is also an emerging readout for recording metabolic activity on biofilm that records biofilm's surface activity.^[44]^ Nevertheless, these imaging techniques may require time‐consuming staining of the biofilm components (bacteria or EPS), special equipment, and highly trained staff members. Because of this, they are not applicable in clinical settings.^[8c]^ Biofilms can also be detected by microscopic techniques alone, which results in frequent false‐negative results because bacteria tend to be patchy and dispersed rather than confluent, particularly on biotic surfaces.^[45]^ While the methods described above have offered advances in biofilm detection, there is still a need for precise and reproducible biofilm detection on the wound site. This can be achieved through biosensor technologies that exploit specific characteristics of biofilm. In Table 2 we compare different traditional and sensor‐based methods for wound biofilm detection.

**Table 2:** Comparison of different traditional and sensor‐based techniques for wound biofilm detection.

Method	Technique	Advantages	Limitations	Ref.
Microbiology assays	Standard clinical microbiology culturing methods	⋅ Performed routinely and easily. ⋅ Well‐established standard of operation. ⋅ Clinical standard for detecting infectious pathogen.	⋅ Inaccurate and fails to detect periprosthetic infection in bacteria unable to produce biofilms. ⋅ Often, biofilms are found in deeper tissues.	[46]
				
Molecular assays	Peptide nucleic acid fluorescence in situ hybridization	⋅ Identification of multiple viable but nonculturable (VBNC) states. ⋅ Rapid identification of pathogens based on 16S rRNA (<24 h). ⋅ Peptide nucleic acids do not repel RNA, so they facilitate stronger binding.	⋅ Antigens of the pathogen and the host cannot be distinguished. ⋅ Planktonic bacteria can also possess DNA derived from extracellular bacteria.	[38b]
	16S rRNA PCR	⋅ Can identify multiple VBNC states. ⋅ Rapid (<24 h). ⋅ Can identify pathogens that are difficult to culture.	⋅ Genetic material may also be contributed by nonviable bacteria. ⋅ The sensitivity of the pathogen to antibiotics cannot be determined. ⋅ A bacterial biofilm is difficult to differentiate from planktonic bacteria. ⋅ DNA derived from extracellular bacteria is also found in planktonic bacteria.	[35]
	FRACS; PRADS; PRAPS	⋅ Capable of identifying multiple types of VBNC. ⋅ Pathogen identification within 24 hours by targeting the 16S rRNA gene.	⋅ Genetic material may also be contributed by nonviable bacteria. ⋅ The pathogen′s sensitivity to antibiotics cannot be determined.	[47]
				
Imaging assays	CLSM and SEM	⋅ This method is most reliable for detecting biofilms on biopsy tissues. ⋅ Surface biofilms can be accurately diagnosed using non‐invasive methods.	⋅ Difficult to perform routinely in clinical practice.	[48]
				
Sensors	Bacterial species and wound biofilm EPS sensors	⋅ Rapid method to detect bacteria and biofilm. ⋅ Specific for a particular species of bacteria.	⋅ Unable to detect all the pathogenic bacteria.	[49]
	Environmental parameter sensors	⋅ Rapid and straightforward method (<1 h).	⋅ Unable to detect the type of bacteria and can be influenced by physical and environmental conditions. ⋅ Usually unable to detect the bacterial infection at early stages.	[50]
	Enzymes sensors	⋅ Rapid detection with high sensitivity (<1 h).	⋅ Usually unable to discriminate active/inactive states of the enzyme. ⋅ Unable to monitor dynamic processes.	[51]

Biofilms on wound beds produce quantifiable biomarkers and lend themselves as indicators for a wound's normal or pathological state.^[52]^ Biomarkers are classified into predictive, diagnostic, and indicative. Predictive biomarkers are used to report the likelihood of benefit from treatment. They can be a powerful tool in designing personalized treatment options according to the needs of specific patient populations. Diagnostic biomarkers can be employed to recognize with high precision single or multiple pathogenic bacteria present in infection sites and facilitate the choice of potential treatment, therefore improving clinical outcomes. An indicative biomarker can be used to assess disease progression or response to therapy in real‐time.^[52]^ Table 3 presents different biomarkers in wound biofilms or the infected host body. Apart from specific biomarkers, pH, transepidermal water loss from peri‐wound skin, nutritional factors (e.g., zinc, glutamine, and vitamins), reactive oxygen species, and temperature are other indicators of wound biofilm establishment.^[53]^

**Table 3:** Biofilm‐associated biomarkers.

Biofilm‐associated biomarker	Potential source	Application	Ref.
Alpha defensin	Body fluid	Diagnosis of chronic biofilm‐associated infections.	[54]
miRNA‐200b and miRNA‐191	Plasma	Detection by plasma‐derived microRNA (miRNA) array and qPCR. Used for diagnosing the condition of patients with diabetic foot ulcer.	[55]
CD34^+^/CD45‐dim circulating cells	Plasma or debrided tissue	Serve as predictors of healing outcome in diabetic foot ulcer patients. Identifiable by flow cytometry and immunohistochemistry.	[56]
β‐catenin and c‐myc	Debrided tissue	High levels indicate healing impairment. Identifiable by immunostaining (IHC/IF).	[57]
BMPR, LRIG1, GATA3, and IDR2,4	Debrided tissue	Present at low levels. Demonstrate gene expression status. Can be identified by immunostaining (IHC/IF), microarray and q‐PCR.	[57a]
Matrix metalloproteinase (MMPs)	Wound fluid	Elevated levels of MMPs are correlated with nonhealing in chronic wounds. Can be measured by ELISA or point‐of‐care qualitative measurement device (e.g., WOUNDCHECK™).	[58]
MMPs	Wound fluid	Low levels indicate nonhealing conditions in chronic wounds. Can be measured by ELISA or quantitative gelatin zymography.	[59]
Biofilm‐associated protein (BAP)	Bacterial cells	Many bacterial species have BAP homologs. Participates in the formation of biofilms on bacterial surfaces.	[60]
Quorum sensing	Bacterial cells	New biomarkers for biofilm detection based on changes of the levels of immune markers and immune cell proliferation altered due to the presence of biofilms	[61]
Cellulose	Biofilm matrix components	Detection of biofilms containing uropathogenic *E. coli*.	[62]
Exopolysaccharide	Biofilm matrix components	*Hestiophilus somni* biofilms have been detected using biofilm detection systems.	[63]
Extracellular DNA	Biofilm matrix components	Used to identify the species involved in the polysaccharide analysis and indicate the presence of biofilm.	[64]
Pyocyanin and uric acid	Wound fluid	Potential indicators of infection and wound healing progress.	[65]

There is an increasing body of work on developing sensors that show potential for the precise detection of different wound biofilm‐associated markers and the provision of reliable information about wound status. This field of study has progressed vigorously, and examples abound. The following classifications illustrate recently developed sensors for the detection of different wound biofilm‐associated markers.

### 3.1 Specific Bacterial Species and Wound Biofilm EPS Sensors

The first category of studies used in wound biofilms indicates whole bacteria in the wound site rather than specific biomarkers produced by them.

**Figure 3:**
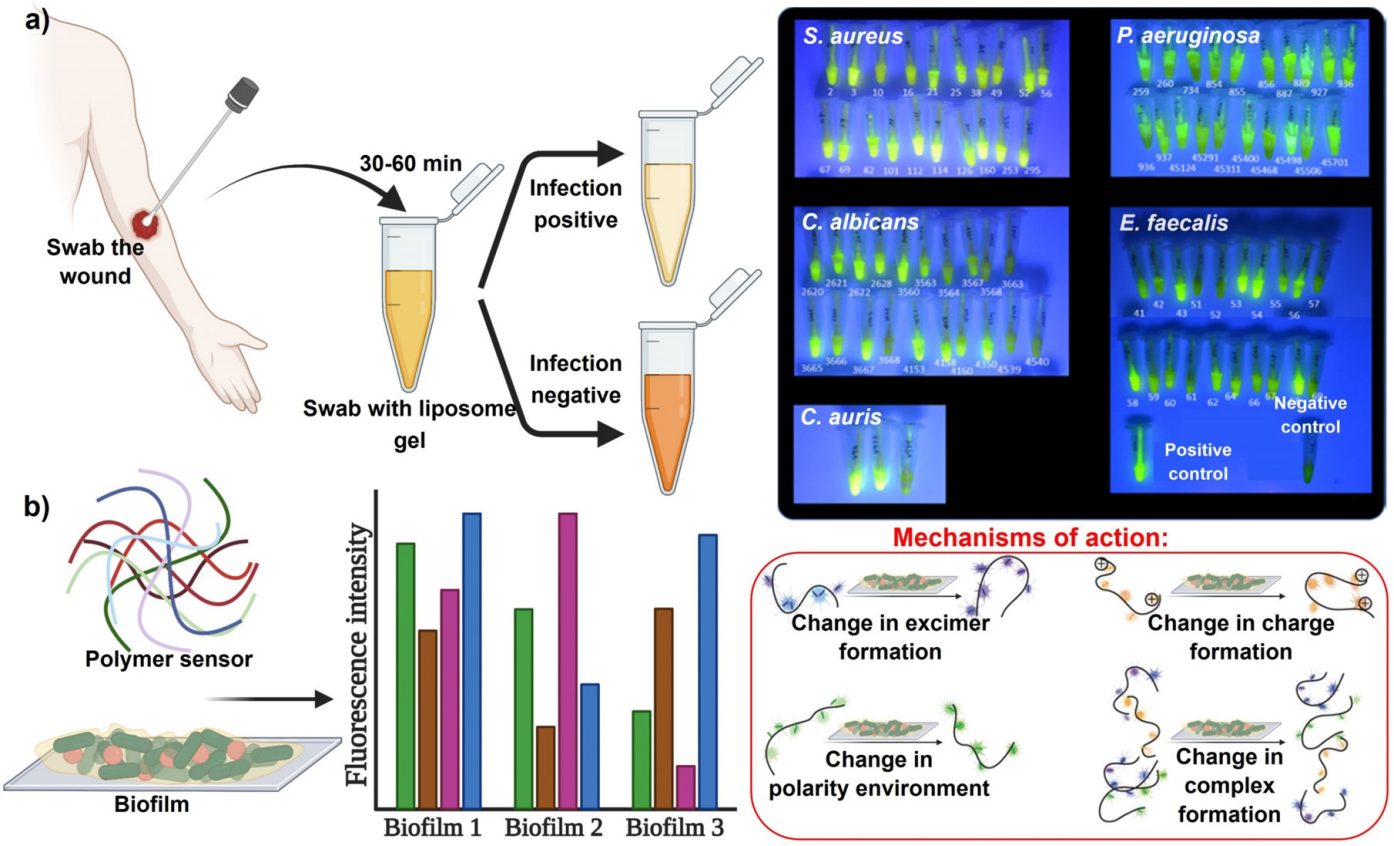
Bacterial species and wound biofilm EPS sensors. a) Illustration of biofilm sensor, with a color change observed in positively infected samples with fluorescence seen from sensor tubes containing different bacterial strains upon UV illumination. b) Fluorescence intensity pattern in connection with biofilms and the mechanisms of interaction of polymers with biofilm matrices. (a) Adapted with permission from ref. [14]. Copyright 2020 American Chemical Society. (b) Adapted with permission from ref. [49b]. Copyright 2019 American Chemical Society.

Thet et al.^[14]^ describe a sensor for detecting skin pathogenic bacteria at an early stage during the formation of biofilms. A fluorescent dye is encapsulated within liposomes. Toxins from infecting bacteria cause liposomes to burst, trigger dye activation, and cause the sensor color to turn from yellow to green, demonstrating infection (Figure 3 a). The biofilms were swabbed and mixed in the liposome suspension, and the colorimetric response characterized the population density of pathogens in the biofilm model. Ngernpimai et al.^[49b]^ synthesized a sensor made of poly(oxanorborneneimide) (PONI) polymers for rapid identification of biofilms. The interactions between these polymers with the biofilm matrix caused differential fluorescent profile changes, providing a species‐based characterization of the biofilm (Figure 3 b). PONI was incorporated into two elements, the recognition element based on cations and one sensitive transducer. Selective binding is an important characteristic of the cationic recognition elements. From a single‐well measurement, each of the PONI polymers provided four characteristic excitation/emission peaks and two effective FRET signals. This sensor shows the ability to discriminate between bacterial species, three of which are pathogenic clinical isolates: *P. aeruginosa*, *Bacillus subtilis* (*B. subtilis*), *E. coli*, *Enterobacter cloacae* complex, and methicillin‐resistant *S. aureus*.

**Figure 4:**
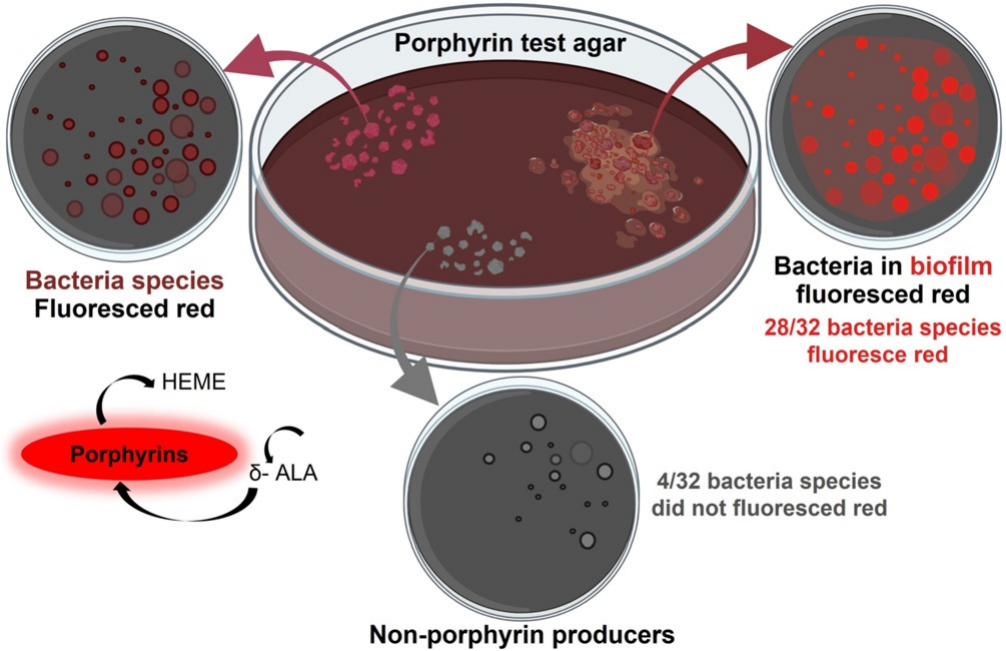
Bacterial species and wound biofilm EPS sensors: schematic representation of the porphyrin production in 32 bacterial species. Under violet light illumination, 28 of 32 bacterial species emitted red fluorescence, while the four known non‐porphyrin‐producing species did not produce the signal. Red fluorescence was observed from porphyrin‐producing bacterial species grown in a biofilm.^[66]^

Jones et al.^[66]^ focused on the visualization of polymicrobial populations based on porphyrin fluorescence. In this study, 32 bacteria and 4 yeast species were plated on agar and tested for red fluorescence. According to the findings, 28 of 32 bacteria studied and one in four yeast species and monomicrobial biofilms produce red fluorescence when they produce porphyrin (Figure 4). Thus, porphyrin production is a primary biological source of red fluorescence, imaged by fluorescence imaging upon excitation with violet light. These sensors offer precise detection for pathogenic bacteria and particularly for chronic nonhealing infected wounds.

### 3.2 Sensors to Indicate Environmental Parameters (e.g., pH and Temperature)

**Figure 5:**
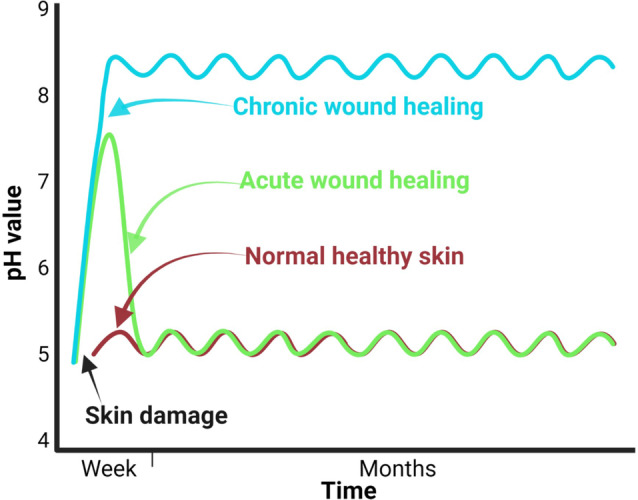
Sensors for wound environmental parameters: Differences in pH between healthy skin, acute wounds, and chronic wounds can be discerned by time courses.

**Figure 6:**
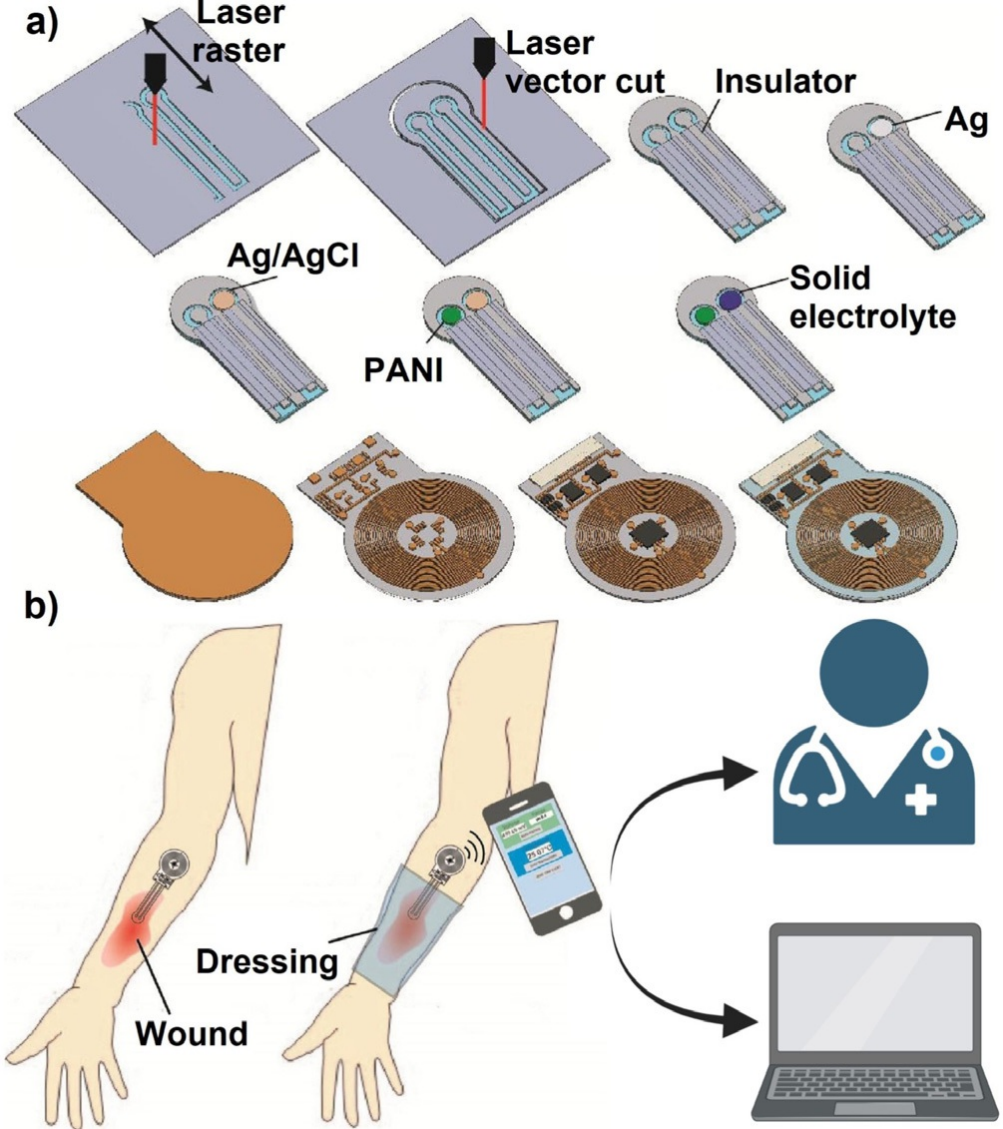
Sensors for wound environmental parameters: a) Fabrication of a pH sensor on an ITO electrode. b) Schematic of the integrated and flexible NFC communication system for measuring wound pH. (a) and (b) Adapted with permission from ref. [72]. Copyright 2018 Elsevier.

Different studies have demonstrated that the pH profiles of chronic, acute, and healthy skin differ significantly.^[67]^ Chronic wounds have an alkaline pH, whereas healthy skin has a slightly acidic pH (Figure 5).^[68]^ When an infected wound has a high pH, it inhibits microorganism growth and invasion. This pH change affects matrix metalloproteinases, fibroblast activity, keratinocyte proliferation, microbial proliferation, and immunologic reactions in the wound.^[69]^ Since pH changes can modulate biological and biochemical processes in wound healing, various pH sensors have been developed to monitor wound status. Different methods are available to measure wound pH, including optical and electrochemical approaches.^[70]^ The wavelengths of absorption and emission of a pH‐sensitive dye used in optical methods are in the visible range.^[71]^ Electrochemical pH sensors mainly measure the pH‐sensitive potential, current, or impedance.^[71]^ In a study by Rahimi et al.,^[72]^ a potentiometric pH sensor was developed for wound infection detection. The wound model was infected with Gram‐positive cocci, *Staphylococcus epidermidis (S. epidermidis*). In addition to their high sensitivity, the sensors are optically transparent, so the tissue beneath the sensor can be imaged. pH changes were monitored successfully by the sensor in simulated in vitro wounds. A laser ablation process was used to produce transparent and flexible electrical substrates from commercial indium tin oxide (ITO) films (Figure 6 a,b).

**Figure 7:**
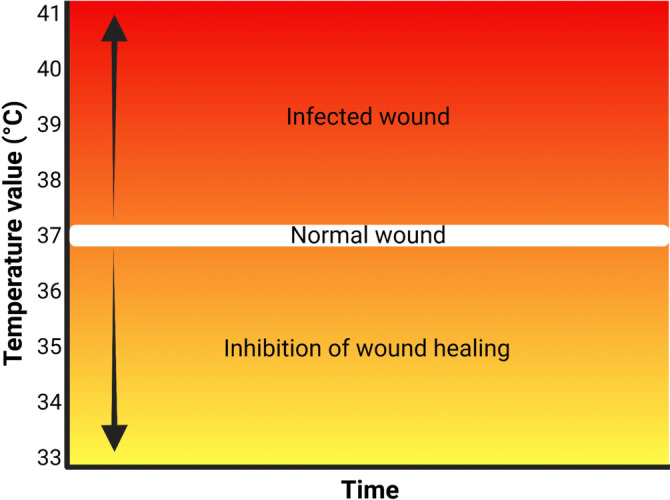
Sensors for wound environmental parameters: Differences in the temperature of normal and infected wounds.

Wound inflammation and infection are closely linked to wound temperature.^[73]^ It is possible to predict infection by observing abnormal wound temperature changes before any secondary symptoms appear.^[74]^ As such, temperature is an important indicator of wound biofilms. With the emergence of flexible electronics, different innovative wound dressings with integrated temperature sensors have been designed to provide precise real‐time information about the wound environment.^[15a]^ The local temperature of wounds lies between 33 °C and 41 °C (Figure 7).^[75]^ Wound repair is hampered when the temperature of the wound is less than 33 °C.^[76]^ Local wound temperatures may exceed 37 °C due to local congestion and inflammation; however, sudden increases in wound temperatures are an indication of infection.^[77]^

Furthermore, higher local wound temperatures were associated with a greater risk for Gram‐positive infection. For instance, Zhang et al.^[78]^ showed that wounds with *P. aeruginosa* and *K. pneumoniae* were about 37.5–38 °C, while wounds with *S. aureus* were 38.5 °C.

**Figure 8:**
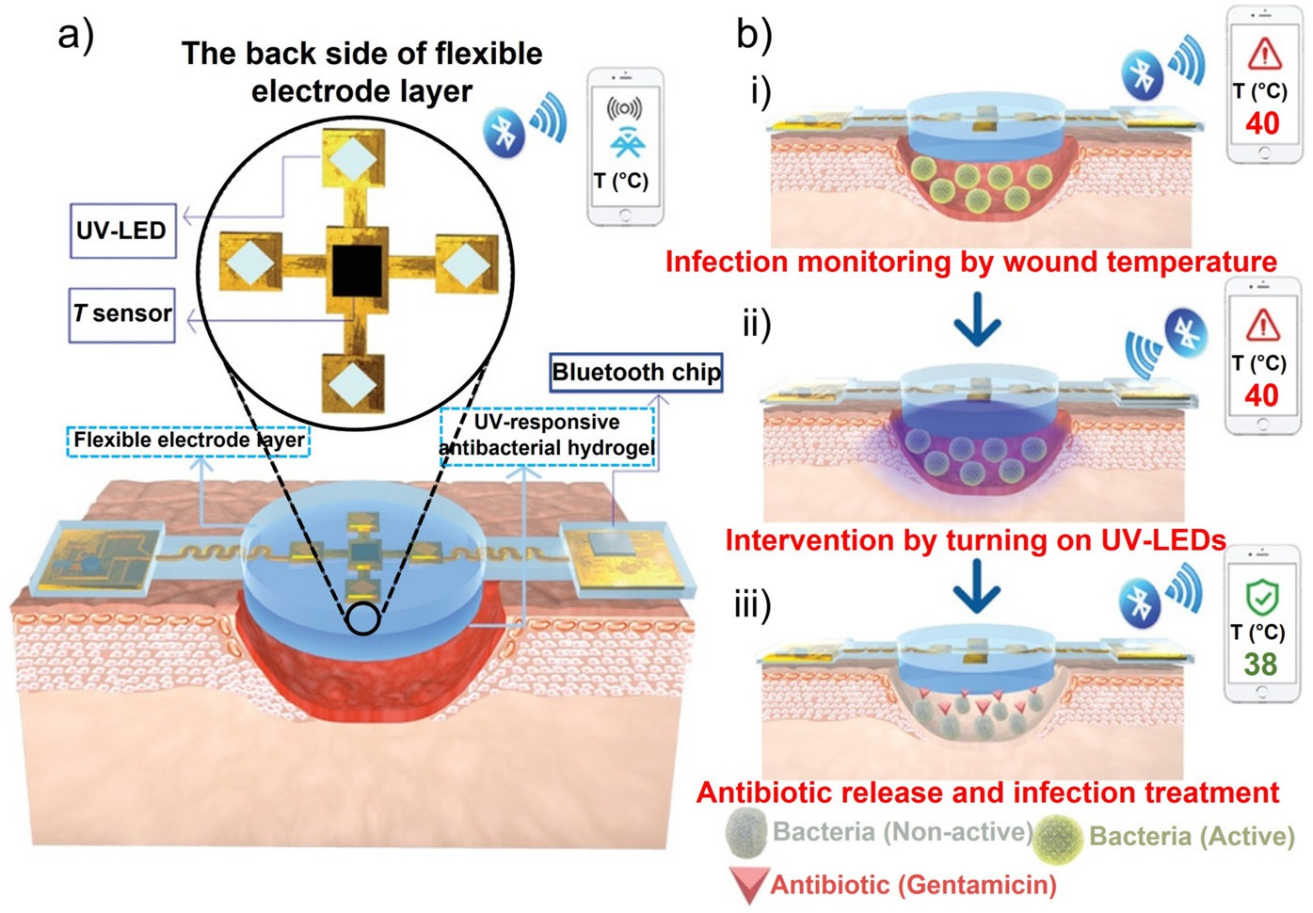
Sensors for wound environmental parameters. a)Construction and working principle of an intelligent dressing, incorporating flexible electronics for wound monitoring. b) Integrated system for monitoring wounds infected with bacteria. (a) and (b) Adapted with permission from ref. [79]. Copyright 2020 Wiley Online Library.

Pang et al.^[79]^ developed a double‐layer structure including polydimethylsiloxane‐encapsulated flexible electronics integrated with a temperature sensor and ultraviolet (UV) light‐emitting diodes (upper layer) and a UV‐responsive antibacterial hydrogel (lower layer). Through the use of an integrated temperature sensor, wound temperatures were continuously monitored and transmitted in real‐time to a smartphone via Bluetooth communication. UV‐LEDs were used to control the release of antibiotics in situ (Figure 8 a,b). Tissue compatibility, high sensitivity, and durability were all device features. Also, the results in vivo in a model of infection in Bama mini pigs showed that the device could detect infection at an early stage and provide an indication of treatment.

Recently, the integration of flexible sensors in a bandage or so‐called innovative wound dressing has attracted much attention. Smart bandages have been engineered to provide precise real‐time information about wound conditions, including pH, temperature, moisture, and oxygen, to personalize individual patients’ clinical treatment.^[15a]^ Smart bandages have been reviewed in detail by Derakhshandeh et al.^[80]^ Although sensor‐integrated wound dressings can provide information about the wound environment, some detectable symptoms may stem from other reactions in the body, leading to an incorrect diagnosis. Multiple sensors have been incorporated simultaneously in wound dressings to provide orthogonal information from various wound parameters to counteract this problem. For instance, Mostafalu et al.^[81]^ designed an automated, flexible wound dressing incorporating potentiometric pH and temperature sensors to provide information on both bacterial infection type and inflammation level. In addition, a thermo‐responsive drug carrier was deployed to provide a controllable drug release system in response to temperature changes. The integration of more than one sensor can give even more accurate information about the wound environment and wound biofilm formation status. However, engineering and integration of a multitude of sensors are still too challenging to be economically viable at present.

### 3.3 Enzyme Sensors

Various studies have demonstrated the role of enzymes, precisely that of matrix metalloproteinases (MMPs), in the healing process of chronic wounds. Metalloproteinases are a group of endopeptidases classified according to their primary catalytic substrate: collagenases, gelatinases, matrilysins, stromelysins, and membrane‐type MMPs.^[82]^ There are more than 20 structurally related zinc‐dependent endopeptidases in the MMP family.^[83]^ Some of these enzymes are excessively released and activated in chronic cutaneous wounds, which lead to long‐term healing. Extracellular material can be digested by MMPs, allowing an influx of reparative keratinocytes, fibroblasts, and endothelial cells. For a wound to heal, MMPs should be at an appropriate level and in the correct location for a precise duration of time. TIMPs (tissue inhibitors of metalloproteinases) are generally produced in excessive amounts during wound healing, and their levels are concomitantly downregulated, resulting in decreased production of MMPs. In response, the balance between MMP and TIMP is altered.^[84]^ MMPs are chronically elevated in chronic wounds, and TIMPS are reduced, such that aberrations in their ratios can serve as potential diagnostic biomarkers to detect wound biofilms. In two different recent review studies by Kirchhain et al.^[85]^ and Lei et al.,^[86]^ different approaches were reviewed to quantify the level of MMPs as a marker of many pathological conditions, together with their strengths and weaknesses. Table 4 provides a summary of different technologies for MMP detection.

**Table 4:** Methods for the detection of matrix metalloproteinases (MMPs).

Detection technique	Advantages	Limitations	Ref.
1. Current methods			
Zymography	Separates different forms of specific MMPs. Active and pro‐enzymes are detectable in parallel	Analytical laboratory necessary, limited substrate choice.	[87]
Immunoassays: ⋅ Enzyme‐linked immunosorbent assay	Sensitive and quantitative; mature technology	No discrimination of active/inactive states of the enzyme. Specificity is limited to just one analyte. Limited shelf‐life of antibodies required for the assay.	[85, 87]

2. Sensing technologies			
Molecular probes: ⋅ Activity‐based probes ⋅ Substrate‐based probes ⋅ Antibodies and other affinity‐based probes	Suitable for applications in vivo	Cannot be used to monitor dynamic processes, and the synthesis of probes can be complex.	[88]

3. Sensors			
Electrochemical sensors: ⋅ Voltammetric ⋅ Impedimetric ⋅ Capacitive	Fast and easy detection; low cost	Electronic interference can limit quantifiability.	[89]
Optical sensors: ⋅ Fluorescence/luminescence ⋅ Reflectometric ⋅ Surface plasmon resonance	Very sensitive and not affected by electronic interference	The availability of light sources can influence measurement. Reporter molecules may be subject to photobleaching	[90]
Other sensors: ⋅ Field effect transistors ⋅ Quartz crystal microbalance	Facile miniaturization and mass production.	Limited test data are available for actual samples. Sensitivity has so far not been reported in depth.	[91]

## 4 Wound Biofilm Therapy

Wound biofilms can pose severe challenges for therapeutic treatment. Chronic or hard‐to‐heal wounds are becoming a “silent epidemic,” affecting up to 2 % of the population in the middle‐to high‐income countries.^[92]^ Prevalence can reach between 3 and 5 % in the senior population as the wound healing processes are slowing down.^[93]^ With an estimated annual cost of USD 20 billion in the United States alone, chronic wound management also has a massive economic impact, burdening patients and healthcare systems.^[94]^ There are few treatment options for clinicians against biofilm infections that efficiently disrupt biofilms without being toxic to the host tissue. Hence, alternative treatment is urgently needed.

**Figure 9:**
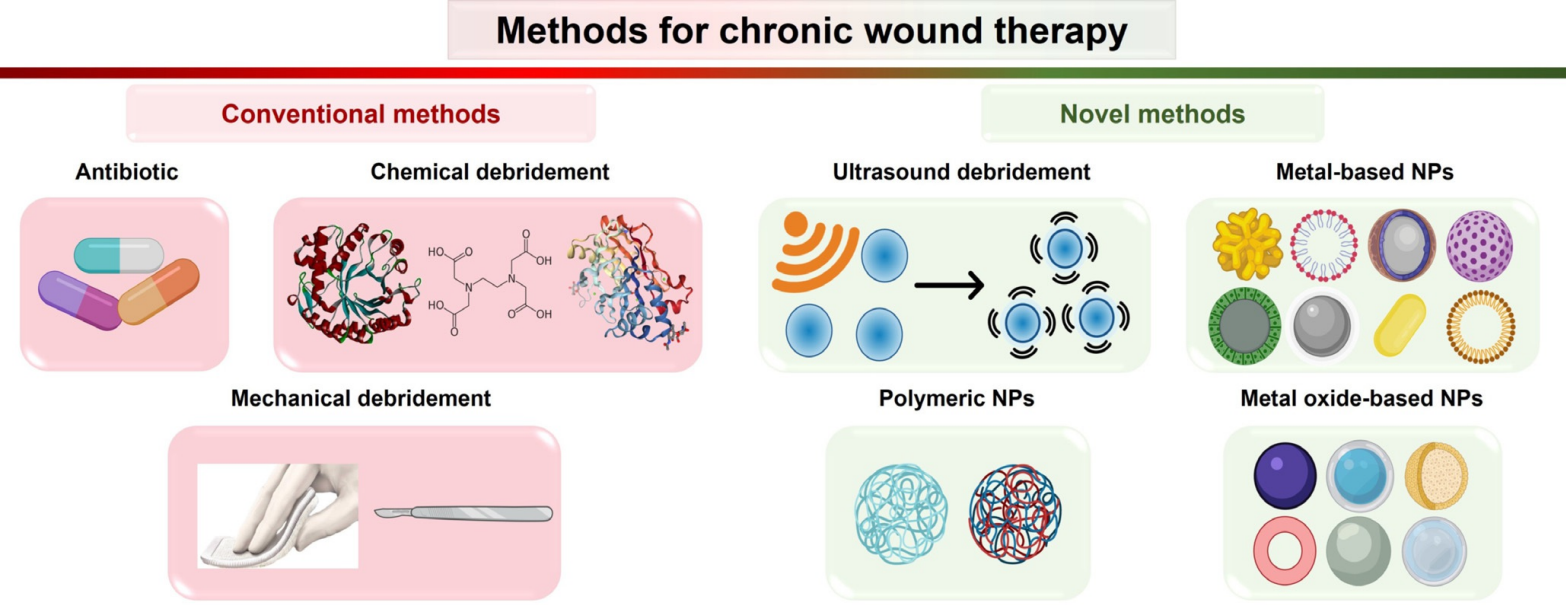
Methods for therapy of chronic wound biofilm.

Mature biofilms are intractable to treatment. Their resistance to antimicrobial agents, disinfectants, and the host's immune system can be up to 1000 times higher.^[95]^ The current standard of care (SOC) for wound biofilm includes frequent biofilm eradication through conventional treatments. The biofilm eradication comprises physical and chemical debridement, and application of topical and systemic antimicrobials and dressings (Figure 9).^[96]^ Here, we review recent developments on the novel treatments based on ultrasonic debridement and nanotechnology, which are more specific and more effective against chronic wound biofilms.

Chronic wound infections caused by biofilms are minimally treatable with topical and systemic antibiotics currently available.^[97]^ EPS acts as a protective barrier inhibiting diffusion and penetration of antimicrobials, resulting in slower or incomplete penetration.^[98]^ It is also possible that antimicrobial agents react chemically with the extracellular components of the biofilm, rendering them ineffective, or they can stick to the anionic polysaccharides without reaching the target bacterium.^[99]^ A growing movement is under way to find non‐antibiotic alternatives to antibiotics due to the emergence of drug‐resistant bacterial strains.^[100]^

### 4.1 Biofilm Eradication

Mechanical debridement or sharp debridement of wounds can interrupt biofilm growth and cause faster wound healing.^[101]^ However, this process can cause damage to healthy skin tissue and the release of bacteria in the area of the wound, which can cause secondary infections. A significant disadvantage of mechanical debridement is that it is often not enough to eradicate all bacteria. The development of resistant biofilms after debridement indicates impaired treatment efficacy and delayed wound healing.^[102]^ Another major disadvantage to sharp debridement is that it causes pain and discomfort to the patient, decreasing compliance and treatment effectiveness.

EPS from biofilms can also be chemically degraded. Manganese and iron are essential for the metabolism of bacteria and the bacterial cell wall structure. Calcium and magnesium crosslink polysaccharides within EPS.^[103]^ The competition for these ions and their removal (chelation) will affect biofilm formation. There is a wide range of metal chelating agents. However, biocompatibility and safety considerations limit their application to polyanions, such as phosphates and citrates, and ethylenediamine tetraacetic acid (EDTA). One of the most common anti‐biofilm agents is EDTA, which has the best calcium and magnesium ion affinity.

A number of extracellular proteins, such as structural proteins and enzymes, may even be present at higher concentrations than polysaccharides within the biofilm matrix. By connecting cells to the extracellular matrix, structural proteins stabilize biofilm architecture.^[104]^ Polysaccharides (e.g., dispersin B), matrix proteins (e.g., proteases), and eDNA (e.g., DNases) are all degraded by enzymes.^[104]^ Recent research used a hybrid method of combining proteases, antiseptics, and EDTA to inhibit the growth of biofilms of *S. aureus* and *P. aeruginosa* in chronic wounds.^[105]^ The destabilizing effect of EDTA comes from chelating cations and blocking the activity of matrix metalloproteases.^[106]^ In addition, it potentiates the action of antiseptics when combined with enzymes, making them more effective when administered at lower doses.^[105]^ Lefebvre et al.^[105]^ reported the broad‐spectrum effect of several bacterial strains using non‐specific enzymes. The combination treatment with synergistic effects significantly reduced bacterial viability. However, there is a need for efficient methods of delivering the molecules into the biofilm.^[105]^

#### 4.1.1 Ultrasound Debridement

**Figure 10:**
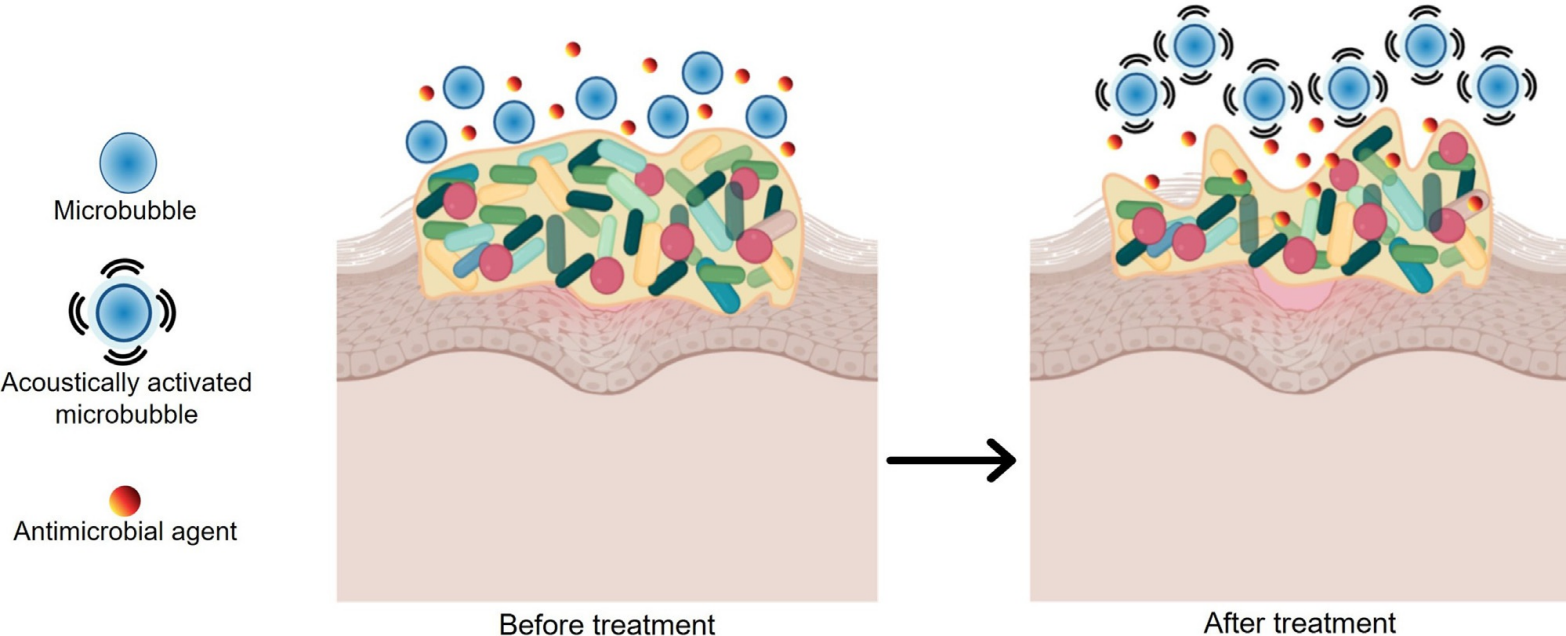
Concept of ultrasound‐mediated microbubble therapy. The microbubbles are acoustically activated after they are delivered and antimicrobials are applied (before treatment) to disrupt the biofilm and allow for the penetration of antibiotics (after treatment).

Ultrasound debridement has been reported to treat wounds infected with biofilms and potentiate antibiotics while promoting the healing process.[^8b^, ^107^] Researchers have observed bacterial counts and wound size decreasing after ultrasound debridement for diabetic foot ulcers and lower‐extremity wounds.[^107d^, ^108^] The advantages of ultrasound debridement are that it is non‐invasive, less painful, and less invasive than sharp debridement.^[109]^ There have been promising developments in the use of ultrasound‐induced micro/nanobubbles in ultrasound‐mediated therapy. Activating nanobubbles and microbubbles with acoustic waves delivers drugs and mechanically disrupts biofilms at the same time (Figure 10).^[8b]^ Gas‐filled bubbles may have various architectures but commonly include a shell comprising polymer, surfactant, protein, or phospholipid encapsulating a gaseous core.^[110]^

The acoustic response to ultrasound stimulation relies on the mechanical stability of the bubbles, which can be controlled by their composition and size.^[111]^ So the size of bubbles plays a key role in acoustic properties, drug loading capacity, longevity, and ultimately, the safety of bubbles in in vivo application.^[112]^ During long‐term storage, microbubbles generally become bigger, with a diameter distribution ranging from 1 to 10 μm.^[113]^ Nanobubbles, on the other hand, vary from 50 nm to 1 μm in size, with a stable range of 50 nm to 300 nm.^[114]^

The size of nanobubbles is determined by the amount of gas dissolved in the solution. Low concentrations of gas result in smaller nanobubbles, whereas high concentrations produce larger ones.^[115]^ Microbubble shells commonly contain phospholipids. An interface between hydrophilic and hydrophobic lipid molecules forms a monolayer, allowing the hydrophilic tails to expose themselves to aqueous environments. In contrast, the hydrophobic heads remain in gaseous environments to stabilize the gas core. The permeability of the microbubble shell depends on the length of the acyl chain of a lipid.^[116]^ Lipids with longer hydrophobic chains are more cohesive and pack well,^[117]^ thus preventing gas entry into microbubble shells during storage and increasing stability throughout the therapeutic period.^[118]^ Surfactants can be added to microbubbles and are especially beneficial for biomedical applications.^[119]^

**Figure 11:**
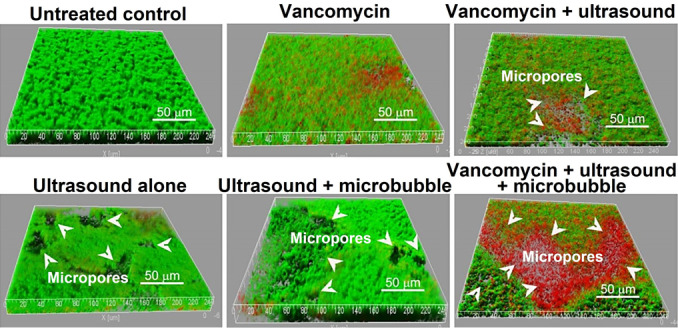
Ultrasound debridement: The LIVE/DEAD viability stain (SYTO9/PI) shows viable cells green and dead cells red in confocal laser scanning microscopy (CLSM) images. Adapted with permission from ref. [124]. Copyright 2011 American Society for Microbiology.

Recent research has focused on adapting composition, size, and fabrication process, and optimizing biocompatibility.[^8b^, ^110b^, ^111c^, ^112^, ^120^] Target applications have mainly been directed towards cancer therapies; however, research on their application for biofilm treatment is increasing. Microbubbles exert biophysical effects by developing localized pushing and pulling forces on cell membranes when subjected to systematic expansion and contraction.^[121]^ Alternatively, fluid can be continuously streamed by oscillating microbubbles (also called cavitation microstreaming). When microbubbles oscillate, divergent (i.e., radial) flows result. When shear stress is increased over nearby cells on a surface that interacts differently with blood flow (e.g., a target tissue), transmembrane pores can form.^[113]^ The streaming flow field can also be exploited to accelerate the shedding of constituents from a microbubble, such as therapeutic compounds.^[122]^ Using this technique, therapeutic material can be deposited (or “printed”) over cell membranes.^[123]^ Using ultrasound‐targeted microbubble destruction, He et al.^[124]^ (Figure 11) demonstrated a significant enhancement of the effect of vancomycin in killing *S. epidermidis* RP62A. Biofilms were treated in vitro for 12 h with vancomycin in combination with ultrasound microbubbles. Following ultrasound exposure, biofilms were cultured for another 12 hours and were found to contain many micropores, and both the film density and viable count of *S. epidermidis* were significantly lower than the controls (Figure 11).^[124]^

**Figure 12:**
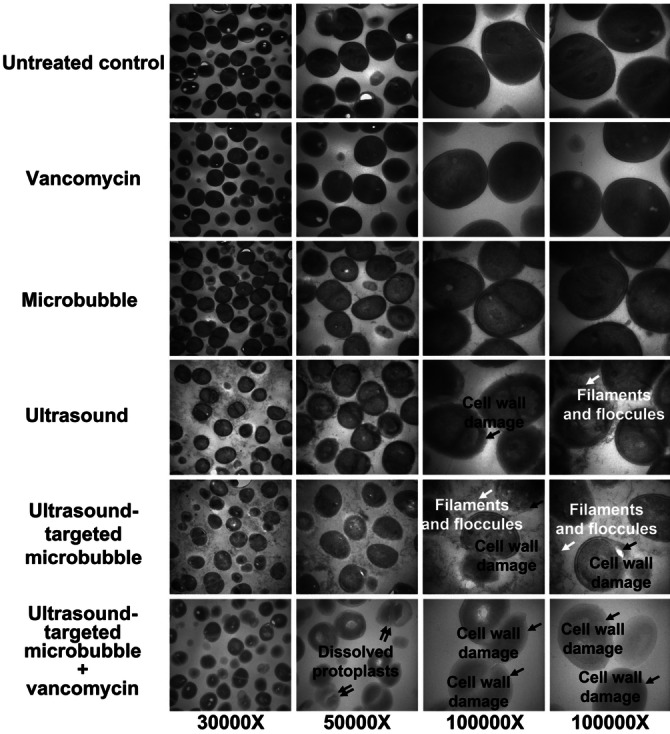
Ultrasound debridement: Image of the ultrastructure of cells in the biofilm recorded by transmission electron microscopy. Adapted with permission from ref. [125]. Copyright 2018 Nature.

Another study by Hu et al.^[125]^ reported that the biofilm produced by a clinical strain of *S. epidermidis* was more sensitive to ultrasound microbubble and vancomycin treatment. Several treated cells showed apparent cell wall damage, and visible cell fragments were observed around damaged cells. (Figure 12).

### 4.2 Nanotechnology

**Figure 13:**
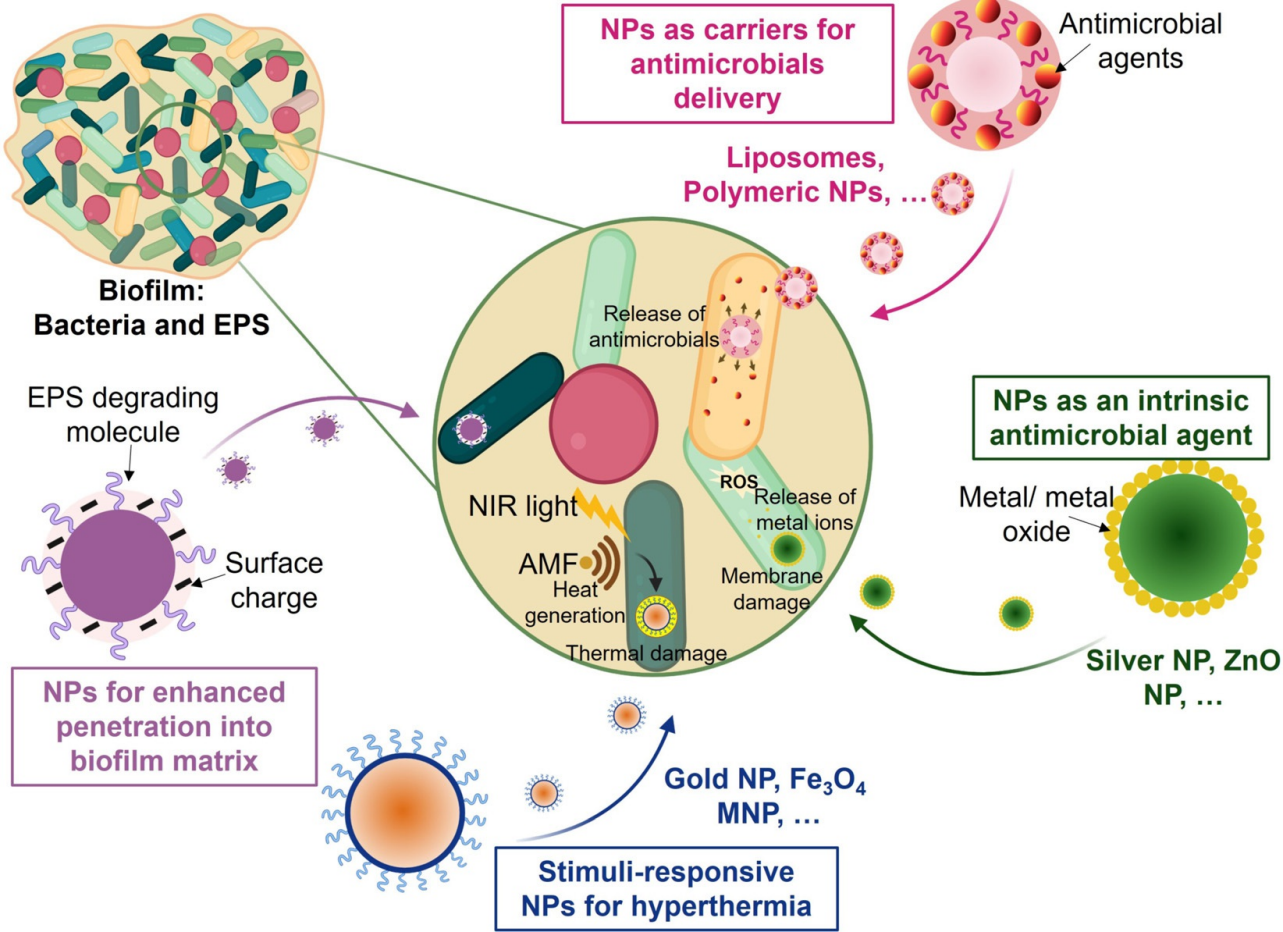
Diagram of the mechanisms of action of various nanoparticle (NP)‐based treatments for biofilm infections.

For chronic wounds, it would be ideal to use a system that is effective against biofilms but does not necessarily involve mechanical disruption. Nanoparticles (NPs) can prevent wound infections caused by biofilms in a new and promising way. Nanoparticle‐based approaches have been developing over the last decade to design nanoparticles with specific chemical and physical properties that prevent and inhibit biofilm infections.^[126]^ Nanoparticle‐based strategies have recently been proposed as potential antimicrobial therapeutics for wounds infected by biofilms. A schematic illustrating how different nanoparticle‐based systems interact with biofilms can be seen in Figure 13. Further details about the design and synthesis of these antimicrobial nanoparticles can be found in recently published review articles.^[127]^

#### 4.2.1 Metal/Metal Oxide Based nanoparticles

Several types of nanoparticles have been shown to possess antimicrobial properties against wound biofilms, including silver, copper, gold, titanium, and zinc. Nanoparticles based on silver have particularly attracted attention. In order to exert their antimicrobial action, silver ions need to interact with sulfhydryl groups.^[128]^ Therefore, they disrupt the integrity of bacterial membranes, respiratory chains, and enzyme activities.^[129]^ As a result, silver ions compromise intermolecular forces and destabilize the biofilm matrix.^[130]^ Wound proteins and other cellular components can readily sequester silver ions, which reduces their bioavailability and antimicrobial effectiveness.^[131]^ In a recent study, Permana et al.^[132]^ reported the selective delivery of silver nanoparticles utilizing dissolving microneedles to improve biofilm skin infection treatment (for more on microneedles see Section 4.2.6). Silver nanoparticles synthesized using green tea extract have been examined as antibiofilm agents against *S. aureus* and *P. aeruginosa* biofilms. The release of silver nanoparticles from microparticles in *S. aureus* and *P. aeruginosa* increased ninefold, demonstrating the selectivity of this approach. It has been shown that dissolving microneedles containing silver nanoparticles improved dermatokinetic profiles more than dissolving microneedles without microparticles. Furthermore, 100 % of the bacterial bioburden was eradicated following administration of this system for 60 hours in an ex vivo biofilm model in rat skin. This study confirmed that silver nanoparticles could be loaded into responsive microparticles for improved antibiofilm performance when delivered with dissolving microneedles.^[132]^

An anti‐biofilm dressing using silver nanoparticles was described in another recent study by Katas et al.^[133]^ Using them may be an effective strategy for reducing wound exacerbations. Silver nanoparticles were produced on a mushroom substrate using chitosan as the stabilizing agent. Gram‐negative bacteria responded more sensitively to nanoparticles with high antibacterial and anti‐biofilm activities. Gelatin hydrogels were formulated from genipin‐crosslinked silver nanoparticles for wound dressings. The antibacterial and anti‐biofilm effect of the hydrogels against *S. aureus*, *B. subtilis*, *P. aeruginosa*, and *E. coli* effectively inhibited the growth of the selected bacteria with the minimum inhibitory concentration of 63 μg mL^−1^. A nanoparticle‐loaded gelatin hydrogel crosslinked with genipin appears to be an effective antimicrobial wound dressing to combat biofilms involved in wound infections.

**Figure 14:**
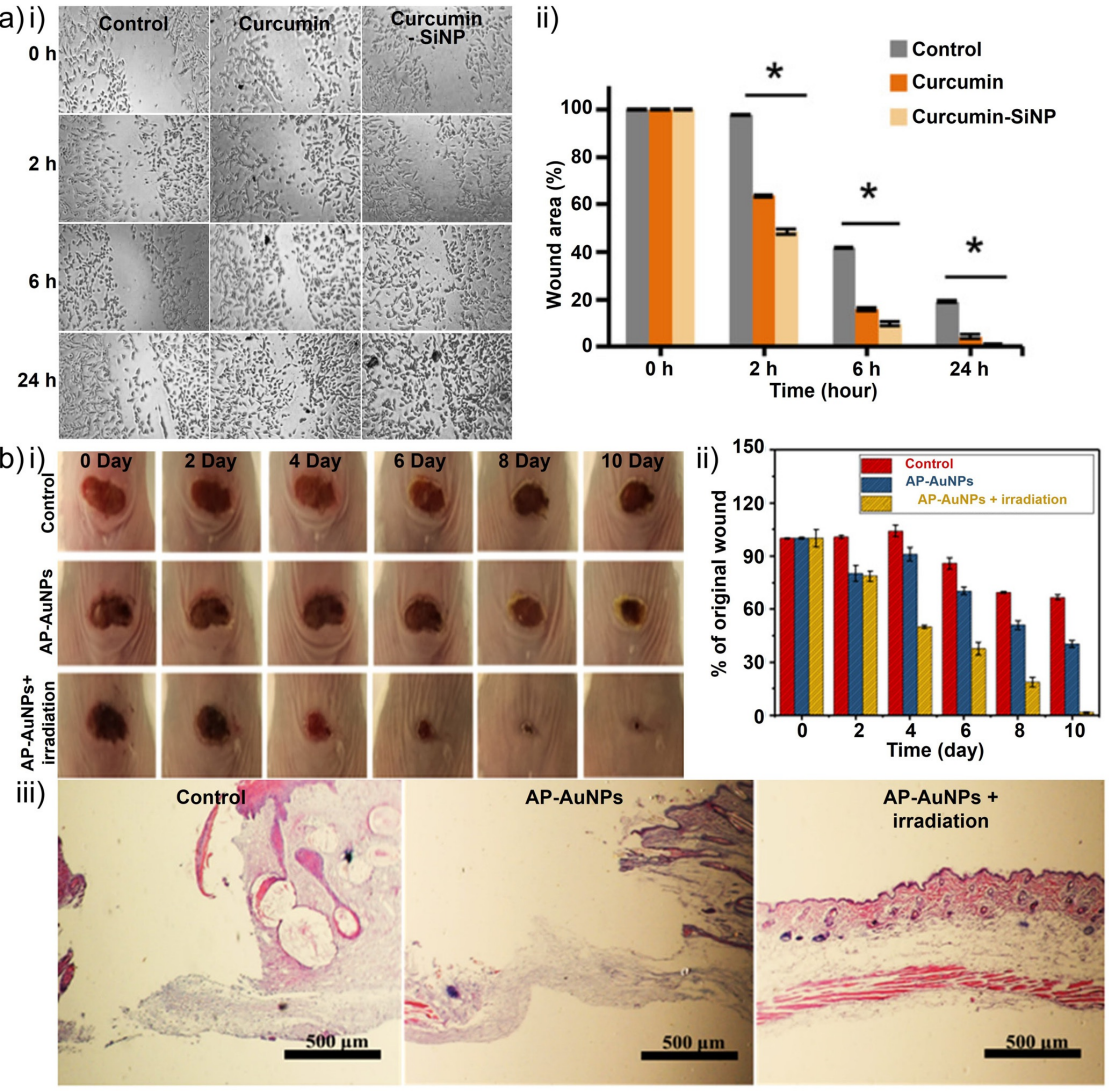
Metal nanoparticles for wound healing: a) (i) Inverted light microscopy images of in vitro scratch wound healing assay of human dermal fibroblast (HDF) cells. (ii) Percentage of wound closure at 0, 2, 6, and 24 h after initiating the treatment of the cells with 50 μg mL^−1^ curcumin and curcumin‐SiNP. *P<0.05 represents a significant difference versus the control. b) Antibacterial and wound‐healing abilities in an animal model. The optical images (i) and corresponding quantification results (ii) of wound changes. (iii) H&E staining of skin tissue from the wounded area on day 10. (a) Adapted with permission from ref. [134]. Copyright 2020 Elsevier. (b) Adapted with permission from ref. [135]. Copyright 2021 American Chemical Society.

Οther nanoparticles have also been proposed in recent studies as antibiofilm reagents. For example, Mirzahosseinipoura et al.^[134]^ examined the photodynamic effect of curcumin silica nanoparticles and free curcumin on planktonic and biofilm forms of *P. aeruginosa* and *S. aureus*. Curcumin–silica nanoparticles were found to decrease bacterial biofilm production and number in planktonic conditions when used as photosensitizers. Additionally, curcumin–silica nanoparticles did not have any significant cytotoxic effect on normal human fibroblasts and showed wound healing properties in an in vitro scratch test. Thus, curcumin–silica nanoparticles could be used to perform antimicrobial photodynamic therapy to treat chronic wound infections caused by multidrug‐resistant bacteria (Figure 14 a).

Qiu et al.^[135]^ investigated the antibacterial properties of gold nanoparticles. AP‐AuNPs (antibacterial photodynamic gold nanoparticles) are synthesized by coupling a photodynamic peptide with poly(ethylene glycol) (PEG)‐stabilized AuNPs. Furthermore, in addition to aqueous and light stability and a remarkable antibacterial effect on *S. aureus* and *E. coli* upon light irradiation, the AP‐AuNPs demonstrated the significant generation of reactive oxygen species (ROS). Additionally, the synthesized nanocomposites inhibited bacterial growth in vitro and prevented biofilm formation. In *S. aureus* infections, photodynamic antibacterial therapy accelerated wound healing, similar to *Staphylococcal* skin infections. The combination of a bactericidal peptide, the photodynamic effect of a photosensitizer, and multivalent clustering on AuNPs results in a maximal antibacterial effect (Figure 14 b).^[135]^

**Figure 15:**
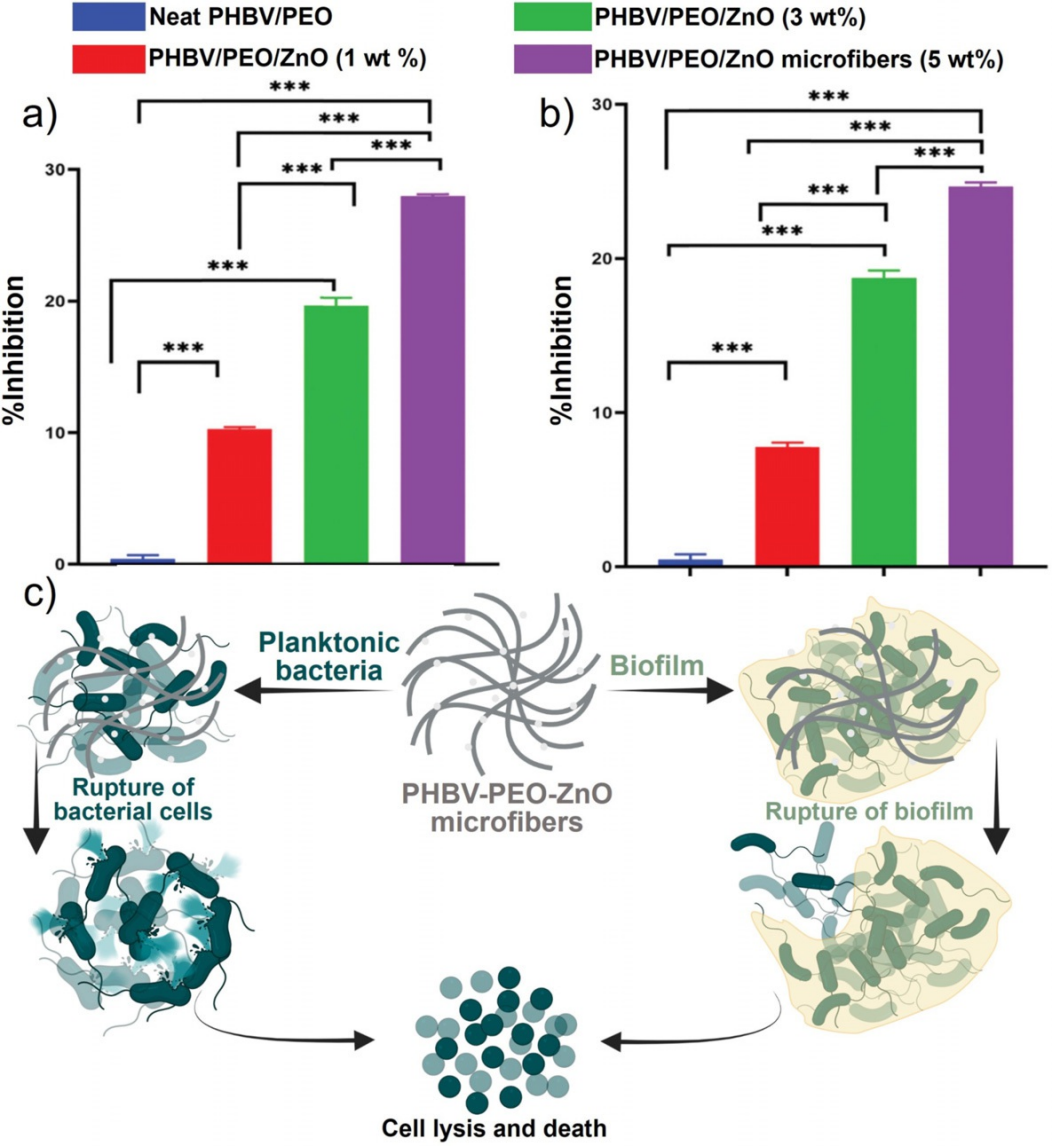
Metal oxide for wound healing: Antibiofilm activity of different polymers against pathogenic bacteria a) *S. aureus* and b) *P. aeruginosa*. c) The mechanism of action of PHBV‐PEO‐ZnO microfibers for antibacterial and antibiofilm treatment. (a–c) Adapted with permission from ref. [142]. Copyright 2020 Royal Society of Chemistry.

Metal oxides are believed to have antibacterial properties, so the development of metal oxide nanoparticles has garnered considerable interest. These oxides include zinc oxide (ZnO), magnesium oxide (MgO), iron oxide, aluminum oxide, and copper oxide (CuO). One of the most widely used nanoparticles is ZnO nanoparticles.^[127a]^ An improvement in wound healing and a reduced bacterial growth rate were observed in a rat wound infection model when ZnO nanoparticles were combined with chitin dressing.^[136]^ In a comparative study, ZnO and CuO nanoparticles were investigated for their respective antimicrobial properties. The antibacterial ability of these products has been proven against Gram‐positive *S. aureus* and *B. subtilis* and Gram‐negative *E. coli* and *P. aeruginosa* bacteria.^[137]^ Additionally, ZnO nanoparticles were found to have antimicrobial properties against biofilms formed by *P. aeruginosa* ^[138]^ and *S. aureus*.^[139]^ Though it is unclear how ZnO nanoparticles work as an antibacterial agent, there has been speculation that hydrogen peroxide production^[140]^ and damage to the cell membrane^[141]^ may be responsible. In a recent study, Mahamuni‐Badiger et al.^[142]^ investigated ZnO NPs incorporated into poly(3‐hydroxybutyrate‐*co*‐3‐hydroxyvalerate) (PHBV)/polyethylene oxide (PEO) microfibers for antibacterial, antibiofilm, and wound dressing applications. A composite of ZnO NPs was prepared in chloroform using PHBV and PEO polymer (4:1) solutions. The antibacterial and antibiofilm activities of the prepared microfibers revealed that ZnO NPs incorporated at different concentrations (1 %, 3 %, and 5 %) displayed different degrees of antibacterial activity against Gram‐positive *S. aureus* and Gram‐negative *P. aeruginosa* (Figure 15 a,b). Results indicated that PHBV‐PEO ZnO (5 %) has a maximum percentage of biofilm inhibition (28.17 %) in *S. aureus* as compared with *P. aeruginosa* (24.51 %). ZnO content in the PHBV‐PEO microfibers increased the percentage of biofilm inhibition. Following the incorporation of ZnO NPs, the PHBV‐PEO‐ZnO microfibers showed excellent hemocompatibility and swelling characteristics. PHBV‐PEO‐ZnO microfibers prepared in this manner were nontoxic as determined by in vitro cytotoxicity assays. An additional focus of this study was the potential effect of PHBV‐PEO‐ZnO microfibers on antibacterial and antibiofilm mechanisms (Figure 15 c).

#### 4.2.2 Polymeric Nanoparticles

**Figure 16:**
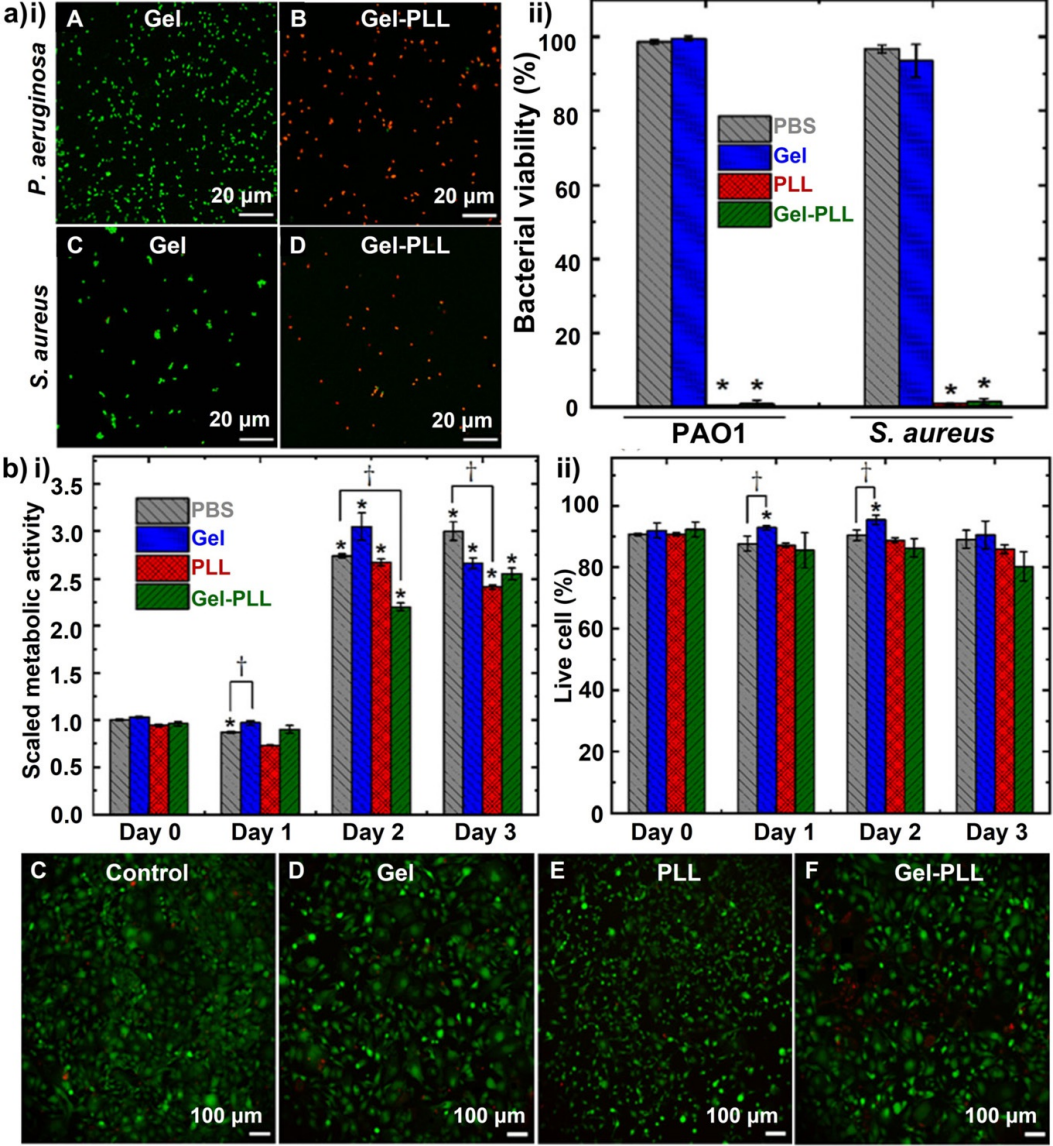
Polymeric nanoparticles for wound healing: a) (i) Representative CLSM images and (ii) quantitative results of bacterial viability after three hours of treatment. b) (i) Quantitative metabolic activity of the cells and (ii) quantitative cell viability results after different treatments. c–f) Representative microscopy images of NIH‐3T3 murine fibroblasts after three days of different treatments. The asterisks on top of the error bars indicate a statistically significant difference (determined using Student's t‐test, *p<0.05). (a) and (b) Adapted with permission from ref. [146]. Copyright 2020 Royal Society of Chemistry.

In addition to offering high structural integrity, storage stability, ease of preparation and functionalization, and controlled release, polymeric nanoparticles are excellent drug delivery vehicles.^[143]^ Polymeric nanoparticles are made from biodegradable polymers or copolymers, in which the drugs can be dissolved, entrapped, encapsulated, or attached. They can be composed of natural, synthetic, and semisynthetic polymers, like gelatin, albumin, alginate, chitosan, poly(glycolic acid), copolymers, PLGA, and PCL poly alkyl‐cyanoacrylate. They have several advantages, including controlled/sustained release, encapsulation degree, enhanced bioavailability, and biocompatibility. As wound dressings or delivery vectors, polymeric nanoparticles (such as chitosan, alginate, cellulose, and hyaluronic acid) exhibit good antibacterial and re‐epithelializing abilities.^[144]^ A significant role for hydrogels in wound healing has been demonstrated among different kinds of polymers. Specifically, wound dressings are utilized to optimize wound bed moisture content by either donating fluid, absorbing excess exudate, or controlling moisture loss.^[145]^ For instance, in a recent study, Tavakolian et al.^[146]^ developed carboxyl‐modified cellulosic hydrogel as the base material for wound dressings. The hydrogel was covalently linked to ϵ‐poly‐L‐lysine, a natural polyamide. The antibacterial efficacy of the hydrogel was tested against two model bacteria, *S. aureus,* and *P. aeruginosa*. The live/dead assay was performed to measure the number of compromised bacteria. Results show that 99 % of the exposed bacteria were killed by the antibacterial hydrogel after three hours (Figure 16 a). Antibacterial hydrogels developed in this investigation are light, have high water‐uptake capacity, and are biocompatible with mammalian cells. As such, they are a potential candidate for wound dressings (Figure 16 b).

Abdalla et al.^[147]^ developed a dual‐antibiotic dressing using gelatin hydrogels incorporated with nanosilver and lactoferrin (Ag‐LTF). An in vitro analysis of the hydrogel's anti‐biofilm and antibacterial properties against *S. aureus* and *P. aeruginosa*, as well as its cytotoxicity, was conducted. Primary wound healing gene expression of HDFs was also examined. Hydrogels formulated with AgNPs and LTF were released at adequate concentrations, demonstrating antimicrobial activity against two bacteria strains. In addition, the cellular functions were not significantly altered by the Ag‐LTF‐loaded hydrogel. These findings indicate that Ag‐LTF and the hydrogels do not affect cell viability, migration rates, or gene expression.

Chitosan is one of the best hydrogel candidates because it is biodegradable, non‐toxic, and antimicrobial. A major benefit of chitosan is that it exhibits antimicrobial activity against both Gram‐positive and Gram‐negative bacteria.^[148]^ Due to its loose cationic nature and low solubility at a pH above 6.5, practical applications are limited. In order to overcome this problem, the backbone chain of chitosan can be modified. Antimicrobial activity was also enhanced by modified chitosan.^[149]^ Chitosan derivatives that are highlighted in the literature include quaternized chitosan,^[150]^ carboxyalkylated chitosan,^[150]^ sulfonated and sulfobenzoyl chitosan,^[151]^ carbohydrate‐branched chitosan,^[152]^ and chitosan‐amino acid conjugates.^[153]^ Another strategy to enhance the properties of chitosan is its combination with metals (oxide).^[154]^ Several studies have used metal oxides (oxides) such as ZnO, TiO_2_, and Ag NPs with chitosan.^[155]^ Examples are mentioned in Table 5.

**Table 5:** Biofilm wound healing: Clinical evidence and treatment options.

Type of wound	Biofilm wound management used	Ref.
1. Physical (mechanical and ultrasound) debridement		
Dehisced	Sharp debridement	[191]
Nonhealing surgical ulcer	Curettage was used to scrape away the underlying film and manage the pathophysiology gently.	[192]
Severely contaminated wounds	Cold atmospheric plasma treatments. Argon‐based Maxium® electrosurgery unit with Maxium® beamer and beam electrode (Gebrüder Martin GmbH + Co. K.G.)	[193]
Venous leg ulcer	Continual debridement and negative‐pressure wound therapy and split‐thickness graft	[192]
Lower limb traumatic wound in a patient with peripheral arterial disease	Biofilm‐based wound care was used. The wound healed in 6 months.	[2]
Venous leg ulcers	Ultrasound debridement patients. Fewer treatments and faster healing than patients treated with sharp debridement.	[109]
Periprosthetic joint infections	Ultrasound sonication for eradicating biofilms; only effective when used in conjunction with antibiotics.	[194]
*S. aureus* biofilm	Acoustically activated nanodroplets with vancomycin decreased biofilm viability and metabolic activity.	[195]

2. Chemical debridement		
Venous leg ulcer	Wound cleaned with sodium hypochlorite between dressing changes.	[192]
*P. aeruginosa* and *S. epidermidis* in vitro	A water‐soluble gel formulation that contains 0.1% EDTA, acetic acid, citric acid, and carbopol.	[196]
Mature biofilm	Tetrasodium EDTA (tEDTA)	[103]
*S. aureus* *P. aeruginosa*	Eugenol as an antimicrobial agent in combination with EDTA.	[197]

3. Antibiotics		
*Acinetobacter baumannii* in both the planktonic and biofilm phenotypes	CZ‐01179. CZ compounds are first‐in‐class series of antibiofilm antibiotics. The name of this class is condensed from the company name, CŪRZA. These compounds are inspired by the antimicrobial potential of naturally occurring peptides and aminosterols, including magainin and squalamine.	[198]
*S. aureus* and *P. aeruginosa* biofilms	Combinatorial effects of antibiotics and enzymes: meropenem and amikacin with the combination of trypsin, β‐glucosidase, and DNase I enzymes	[199]

4. Nanotechnology		
*E. coli* and *S. aureus*	Scaffolds of chitosan + ZnO NPs + silk sericin. Higher antimicrobial activity increased HaCaT cells’ proliferation and viability compared with chitosan/silk sericin/acid lauric.	[155a]
*S. aureus* and *E. coli*	Films of chitosan + polyaniline +montmorillonite + ZnO NPs. High antimicrobial activity against *S. aureus* and *E. coli*.	[200]
Full‐thickness cutaneous wounds	Bilayer composite of chitosan + TiO_2_ NPs. High antimicrobial activity, proper physiochemical, good biocompatibility and faster wound healing.	[155b]
E‐spun mats against *E. coli*	Fiber mats of chitosan + poly(vinyl alcohol) + Ag NPs. Ag NPs improved electrospinnability, decreased the diameter of fibers, and enhanced antimicrobial activity against *E. coli*.	[155c]
*S. aureus* and *E. coli*	Textiles of chitosan + ZnO NPs. High antimicrobial activity. Chitosan + ZnO NPs showed 87% improvement in biocompatibility, and cell viability was steadily decreased after one week.	[201]
*K. pneumoniae*, *P. aeruginosa*, *E. coli*, *B. subtilis*	Multiwalled CNTs showed 82.53 %, 80.98 %, 76.83 %,and 77.41 % biofilm inhibition against *B. subtilis*, *E. coli*, *K. pneumoniae*, and *P. aeruginosa*, respectively.	[202]

5. Combined therapies		
Lower limb wounds, critically ischemic	Sharp and ultrasonic debridement combined with lactoferrin/xylitol, cadexomer iodine, and silver dressings.	[203]
Traumatic chemical burn in a patient with diabetes	Debridement, systemic and topical antibiotics, and silver dressing used, and the patient healed in 12 weeks.	[204]
Peripheral arterial disease	Sharp debridement plus silver carboxymethyl cellulose dressing.	[205]
Highly exuding wounds	Two wounds healed using antibiotics, debridement, and silver carboxymethyl cellulose dressing.	[205]
Patient with diabetes and cellulitis	Antibiotics, debridement plus silver carboxymethyl cellulose dressing.	[205]
Mixed etiologies being given cell‐based therapy	Debridement plus personalized topical gels containing antibiotics and antibiofilm agents.	[206]
*S. aureus*	After 18 h of incubation with the lignin/PVA andlignin/t‐MWCNT/PVA NFs, bacterial growth decreased by 60 % and 69 %, respectively, compared with the control.	[207]
*E. coli* and *S. aureus*	Multiwalled CNTs with the polypyrrole polymer. The anti‐biofilm activity is field‐dependent, reaching a reduction of 40 % for *E. coli* and 90 % for *S. aureus*.	[208]

#### 4.2.3 Liposome Nanoparticles

Nanocarriers protect drugs from degradation, enhance intracellular absorption, offer controlled and sustained delivery, and optimize the location of active compounds.^[156]^ Liposomes are excellent carriers of hydrophilic molecules like hydrophilic peptides^[157]^ and macromolecules.^[158]^ Due to their lipid bilayer structure that mimics cell membranes and is biocompatible, liposome nanoparticles are widely used as drug delivery vehicles.[^158^, ^159^] Hydrophilic drugs can be encapsulated in their aqueous interior, and hydrophobic drugs can be contained in their phospholipid membranes.[^127b^, ^160^] To prevent rejection by the reticulo‐endothelial system^[161]^ and allow penetration through water channels^[162]^ in infectious biofilms, liposomes for infection control should have diameters in the range of 100–200 nm.^[163]^ There are four types of liposomes: cationic, anionic, zwitterionic, and fusogenic. In general, negatively charged bacteria are more likely to react with cationic liposomes.^[164]^ The use of liposomes in conjunction with hydrogels could prevent rapid drug release.^[165]^

**Figure 17:**
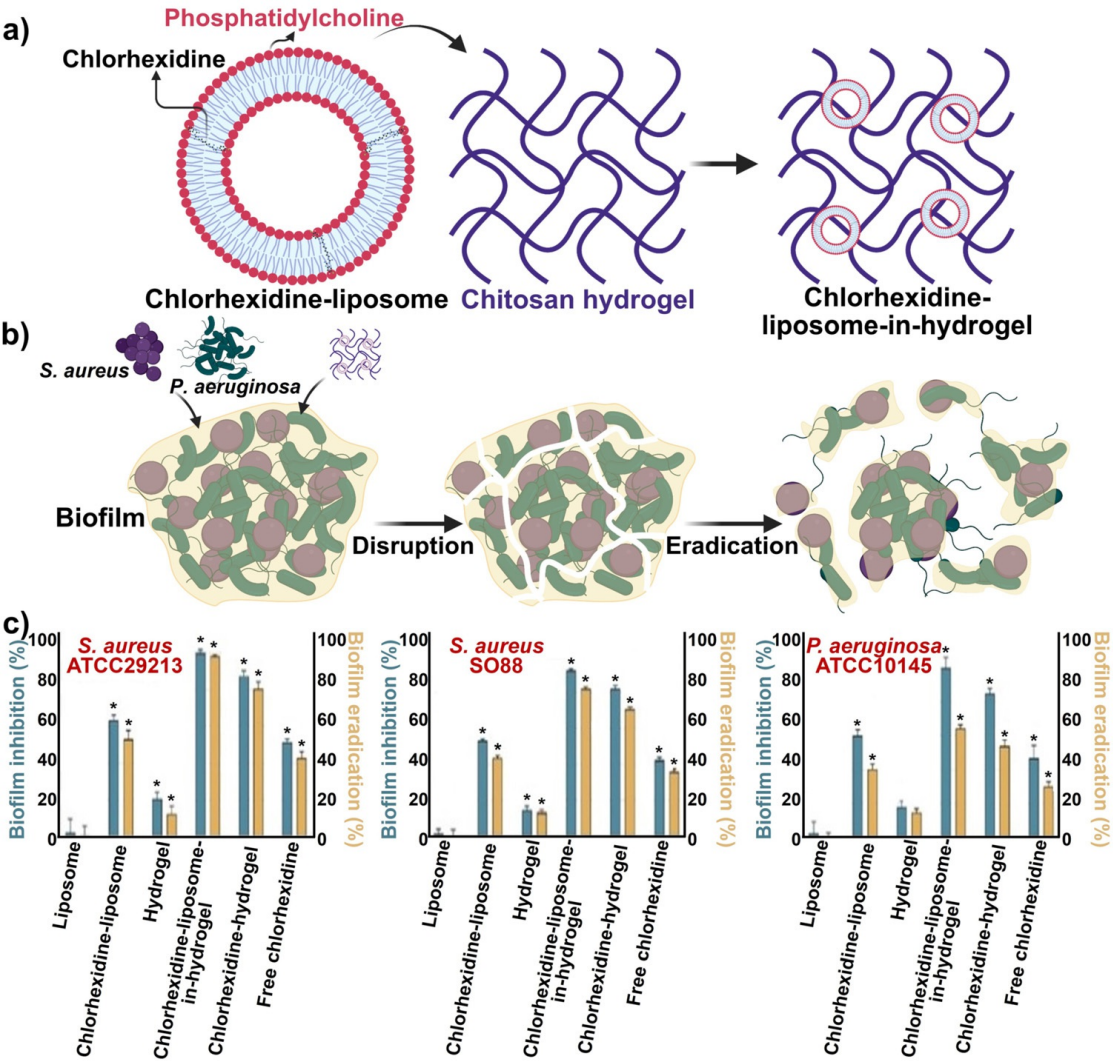
Liposome nanoparticles for wound healing: a) Fabrication of chlorhexidine‐liposome‐in‐hydrogel. b) Effect of the chlorhexidine‐liposome‐in‐hydrogel biofilm leading to disruption and eventual eradication of biofilm. c) Anti‐biofilm activity of chlorhexidine‐liposome‐in‐hydrogel in inhibition and eradication of the biofilm. The results are presented as the mean of three replicates with their respective SD. (*) Significantly different from untreated control (*p*<0.05). (c) Adapted with permission from ref. [166]. Copyright 2021 Elsevier.

Hemmingsen et al.^[166]^ developed a liposomes‐in‐chitosan hydrogel to boost the effectiveness of chlorhexidine for eradicating biofilms in vitro. Electrostatic interactions between negatively charged phospholipids and the positively charged amino groups of chitosan cause chitosan to coat the surface of the negatively charged liposomes.^[167]^ Chitosan is an excellent biopolymer for coating liposomes, as it increases the stability of liposomes and prevents leakage. In addition to increase efficacy, the chitosan coating helps minimize drug release in undesirable locations. It promotes the cellular uptake of the liposomes by cells due to its positive charge.^[167b]^ Figure 17 illustrates the synthesis (Figure 17 a) and effect of chlorhexidine‐liposome‐in‐hydrogel against biofilms (Figure 17 b). In lipopolysaccharide (LPS)‐induced macrophages, chlorhexidine‐liposomes‐in‐hydrogel significantly inhibited nitric oxide (NO) production and reduced the adherent bacterial cells in biofilm by 64.2 %–98.1 %. Chlorhexidine's antimicrobial and anti‐inflammatory effects were improved by chitosan hydrogels (Figure 17 c).

#### 4.2.4 Carbon‐Based Nanoparticles

Carbon nanomaterials are highly biocompatible and exhibit strong antibacterial properties.^[168]^ It is possible to use carbon nanomaterial biomolecules alone or in combination with other materials as antibacterial agents.^[169]^ Carbon dots (CDs) are a new type of nanomaterial that have attracted considerable attention because of their unique properties, including optical properties, good water solubility, low toxicity, biocompatibility, and cell permeability.^[170]^ As a result of their exceptional chemical and photoelectric properties, CDs are great candidates for antibacterial theranostic applications.^[171]^ Recently, Li et al.^[172]^ prepared CDs with gentamicin on ammonium citrate through thermal decomposition. Based on the in vivo wound healing models conducted on the backs of rats infected with *S. aureus*, the CD‐hydrogel showed better skin healing capabilities than the commercially available hydrogels and demonstrated good biocompatibility.

The exceptional mechanical strength, thermal conductivity, photoluminescence properties, and structural stability of CNTs make them an excellent material.^[173]^ Several different therapeutic molecules can be absorbed on the surface of CNTs and transported directly into cells without being metabolized by the body.^[174]^ A coating of CNTs prevents bacterial adhesion and subsequent biofilm formation on medical devices and prosthetic implants.^[175]^ There is evidence that long immobilized nanotubes create unstable substrates as a consequence of their mobility which prevents bacterial settlement and biofilm formation.^[176]^ For instance, He et al.^[177]^ developed photothermal antibacterial nanocomposite hydrogels of Pluronic F127/carbon nanotubes with conductive self‐healing and an adhesive surface. As a result of the addition of CNTs, the hydrogel exhibited promising antimicrobial activity and excellent conductivity in vitro and in vivo.

**Figure 18:**
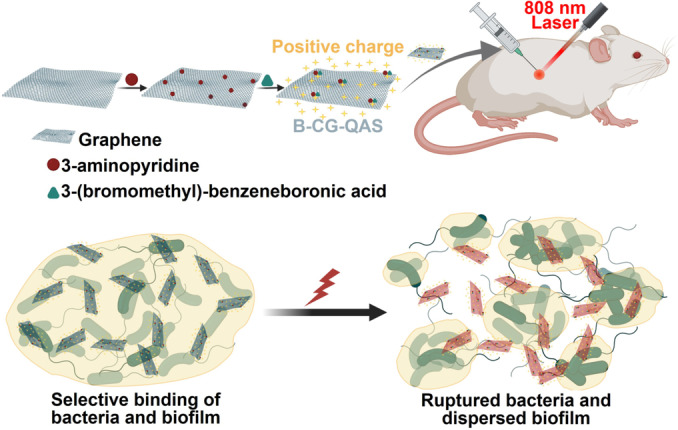
Graphene nanoparticles for wound healing: An illustration of how BCG‐QAS acts against biofilm. Adapted with permission from ref. [181]. Copyright 2020 Elsevier.

Nanostructured graphene and derivatives of graphene have antibiofilm properties due to both the presence of sharp edges and oxidative stress.^[178]^ Indirectly, they inhibit biofilm formation by damaging bacterial cell membranes and causing the loss of proteins, RNA, and other intracellular species when they contact bacterial cells directly.^[179]^ GO has been reported to have antibacterial activity only when a biofilm has reached a specific maturation stage.^[180]^ In a recent study, based on the boronic acid functionalized graphene quaternary ammonium salt (B‐CG‐QAS), Wang et al.^[181]^ reported a new dual‐targeted antibacterial platform. A dual effect of electrostatic adhesion and covalent coupling enabled B‐CG‐QAS to specifically bind to bacteria and their biofilms at the sites of infection caused by Gram‐negative bacteria, resulting in superior targeting ability (Figure 18).

Further improving the antimicrobial effect could be achieved by near‐infrared laser irradiation in synergy with hyperthermia. Moreover, B‐CG‐QAS could be used effectively to treat multidrug‐resistant Gram‐negative bacteria and their biofilms, as well as to speed healing of wounds that are infected with bacteria (Figure 18).

#### 4.2.5 Nanoemulsions

**Figure 19:**
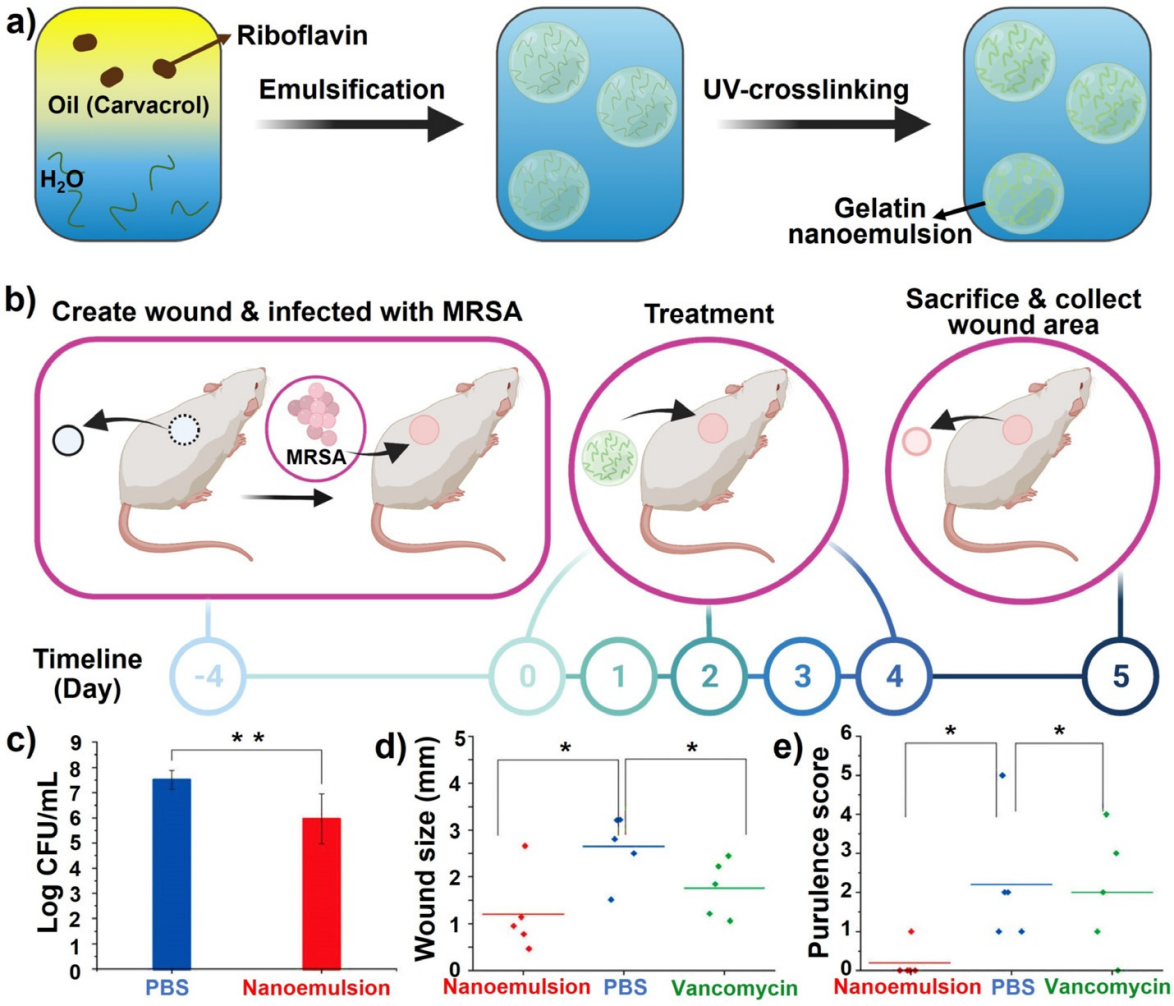
Nanoemulsions for wound healing: a) Schematic representing the fabrication of gelatin nanoemulsions. b) An overview of the biofilm‐associated wound infection model in mice. c) The number of colonies in the infected wounds after treatment with nanoemulsions and PBS. d) The size of the wound on the day of sacrifice. e) Score of purulence at sacrifice day (*, **=*P* values o0.05 or 0.01, respectively). (c), (d), and (e) Adapted with permission from ref. [183]. Copyright 2021 Royal Society of Chemistry.

Nanoemulsions are nanosized emulsions designed to deliver drugs directly and efficiently to target sites under physiological conditions with a long‐term therapeutic effect. Using nanoemulsions as carriers of essential oils could result in lower concentrations being required to achieve equal levels of microbial inactivation compared to conventional emulsions or bulk oils.^[182]^ Both Gram‐positive and Gram‐negative bacterial biofilms were eradicated in vitro by nanoemulsions. Based on a murine wound biofilm model, it was found that nanoemulsions could reduce bacterial loads in wounds and accelerate wound healing. In a recent study, Li et al.^[183]^ reported on a bacterial biofilm in vivo treatment that accelerated wound healing through the use of all‐natural materials. Gelatin was stabilized by photo‐crosslinking with riboflavin (vitamin B2) and carvacrol (the primary component in oregano oil) as the active antimicrobial component (Figure 19 a). The engineered nanoemulsions were demonstrated to have broad‐spectrum antimicrobial activity against drug‐resistant bacterial biofilms in an in vivo murine wound biofilm model. These nanoemulsions show antimicrobial activity, wound healing promotion, and biosafety characteristics to manage wound infections. A murine wound biofilm model was used to evaluate the in vivo activity of the nanoemulsions after their in vitro evaluation. (Figure 19 b). Nanoemulsions significantly reduced the number of bacteria in wounds compared to PBS controls (Figure 19 c). After treatment with nanoemulsions, the wounds were significantly smaller than those treated with vancomycin or PBS (Figure 19 d). The wound beds of mice treated with nanoemulsions had normal‐looking healed wounds and zero purulence scores in contrast to the vancomycin group (Figure 19 e).

#### 4.2.6 Microneedles

Microneedles (MNs) serve as carriers for therapeutic agents, as the MN structure is able to penetrate the top layer (biofilm and cellular debris) and dissolve upon contact with biological fluid thus releasing therapeutics into the wound. In addition to bypassing the stratum corneum without hypodermic needles, MNs deliver drugs more effectively than hypodermic needles as they inject directly into the bloodstream rather than muscle tissue.^[184]^

**Figure 20:**
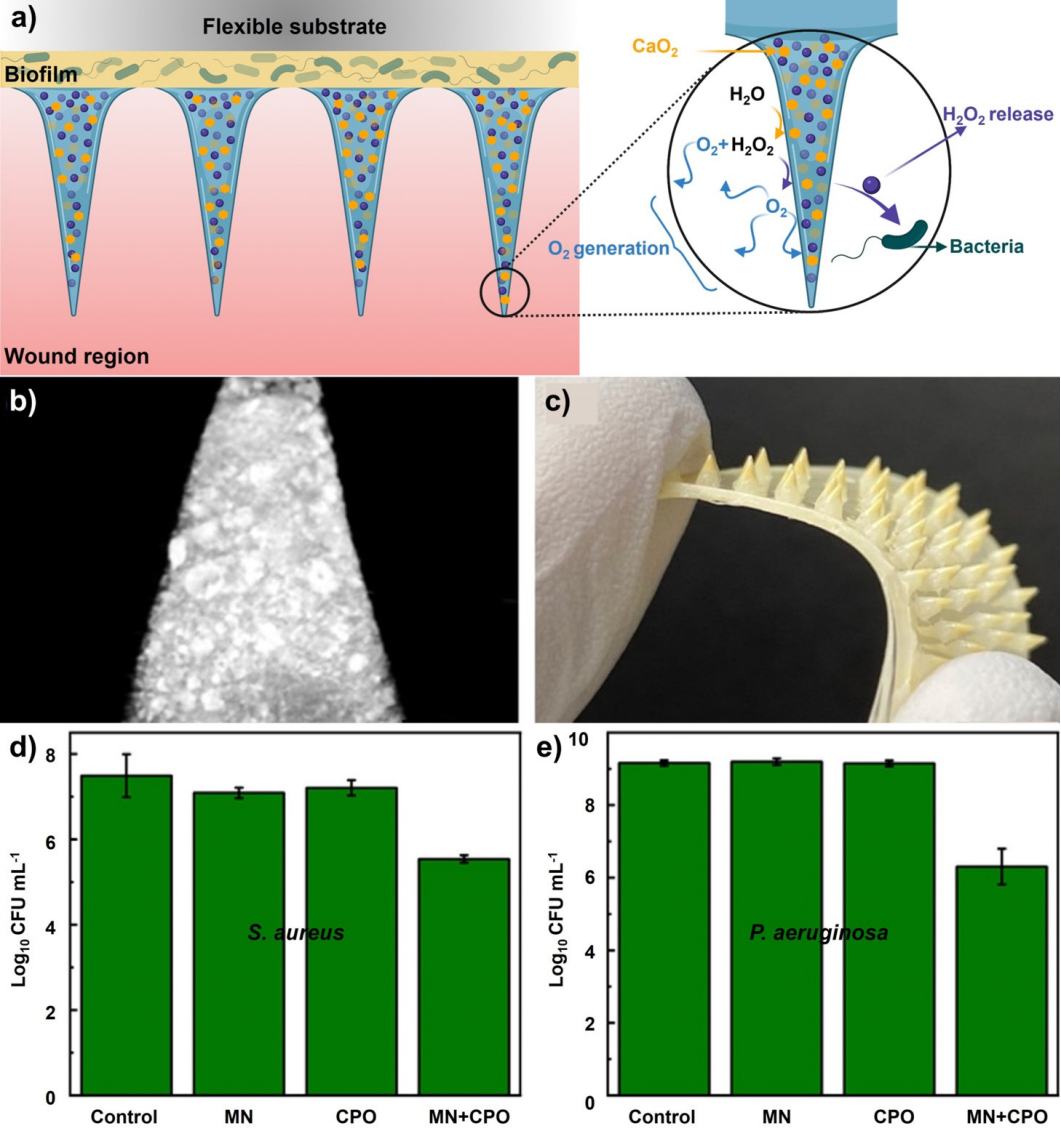
Microneedles for wound healing: a) An illustration of MN arrays of PVP‐CPO embedded on a flexible substrate that are designed to oxygenate wounds and treat biofilm infections. b) Micro‐CT image of one MN loaded with CPO. c) The CPO‐loaded MN array has a high degree of flexibility. Bactericidal studies on 1 week old biofilm: d) *S. aureus* biofilm, and e) *P. aeruginosa* biofilm. (d) and (e) have three conditions of PET (control), MNs without CPO, CPO powder, and MNs loaded with CPO. (b), (c), (d), and (e) Adapted with permission from ref. [185]. Copyright 2021 American Chemical Society.

According to Woodhouse et al.,^[185]^ a polymer composite microneedle array can penetrate physicochemical barriers (such as bacterial biofilms) to deliver oxygen and bactericidal agents to chronic wounds. The microneedles were found to have strong bactericidal effects on both liquid and biofilm cultures of Gram‐positive (*S. aureus*) and Gram‐negative (*P. aeruginosa*) bacterial strains (Figure 20 a–c). Calcium peroxide (CPO) alone did not affect colony number (Figure 20 d,e). By contrast to CPO powder, MNs loaded with CPO significantly reduced bacteria in mature biofilms formed by *S. aureus* and *P. aeruginosa* (Figure 20 d,e). The flexible microneedle array improves the effectiveness of topical oxygenation as well as the treatment of wounds infected with intrinsically antibiotic‐resistant biofilms. Su et al.^[186]^ developed a biphasic scaffold as an antimicrobial delivery system by combining nanofiber mats and dissolvable microneedle arrays. A variety of antimicrobial agents, including AgNO_3_, Ga(NO_3_)_3_, and vancomycin, were electrospun into nanofiber mats, which allowed for sustained delivery. Integrated antimicrobial agents provide direct access to drugs within biofilms through dissolvable microneedle arrays. Combining nanofiber mats with microneedle arrays can deliver multiple antimicrobial agents to wound sites effectively with a combination of nanofiber mats and microneedles.

Apart from the approaches discussed above, there are other examples of emerging technologies that show promise against planktonic bacteria and biofilms, such as nanocomposites (Table 5), DNA nanotechnology^[187]^ micelles,^[188]^ and dendrimers.^[189]^

Each of the biofilm treatment strategies discussed above present advantages and disadvantages. For example, nanoparticles exhibit high stability and ease of production and functionalization but are hampered by potential side effects and cytotoxicity. Ultrasound‐mediated approaches show great promise through the combination of drug delivery and mechanical disinfection. However, the complicated implementation of the technique means that it is not yet clinically viable, and its success is very highly dependent on the size and shape of the wound. Finally, microneedles are safe and can be used to deliver a variety of substances but this approach might be difficult to scale up for use in clinical settings. The next breakthrough in drug delivery for wound infections will likely come from a combination of recent innovations rather than from one single field.

## 5 Conclusion and Perspectives

Microbial control in open wounds is still an unsolved problem in modern medicine. The complexity of biofilms and their increased resistance to traditional disinfection processes make them a very challenging target. Early, accurate sensing of biofilm establishment in the wound can offer opportunities for early intervention, thereby increasing the chances for efficient treatment. Moreover, advances in biofilm sensing have the potential to improve our understanding of the crucial factors that affect the establishment and severity of biofilm infections, thereby enabling the development of more sophisticated treatment options. There has been promising progress in biofilm sensing in recent years, with sensors becoming more accurate and precise. The significant growth of studies on the early detection of biofilms within the past decade indicates the importance and demand for further research and development of such technologies. While most studies on biofilm sensing remain mostly laboratory proof‐of‐concept studies, several in vivo applications have been translated into clinical settings. In order to establish tools that succeed in the laboratory and become widely available, it is crucial that work is continued in this direction.

Eradicating biofilms remains a challenge. Novel approaches have shown promise both in the areas of mechanical and chemical debridement and also through nanotechnology‐based therapies. In the future, the development and establishment of innovative solutions to treat biofilms in open wounds should be an area of significant research focus. The combination of reliable sensing with efficient antimicrobial delivery in biofilms has the potential to provide a much‐needed breakthrough in wound biofilm treatment. Even though great progress is being made on in vitro studies and in vivo on animal models, there is still very limited work on human subjects. However, recent calls for action and the corresponding actions from governments both in the EU and the U.S. promise to incentivize antimicrobial development and translation of new technologies to the clinic, through economic, legislative, and regulatory actions.^[190]^

## Conflict of interest

The authors declare no conflict of interest.

## Biographical Information

*Sorour Darvishi is a PhD student in Chemistry and Chemical Engineering at the Ecole Polytechnique Federale de Lausanne (Switzerland) under the supervision of Prof. Hubert H. Girault. She is also an SNSF visiting fellow at the University of Cambridge and working with Prof. Clemens F. Kaminski. Her PhD projects are mainly focused on the bioimaging of cancer biomarkers and biofilm*.

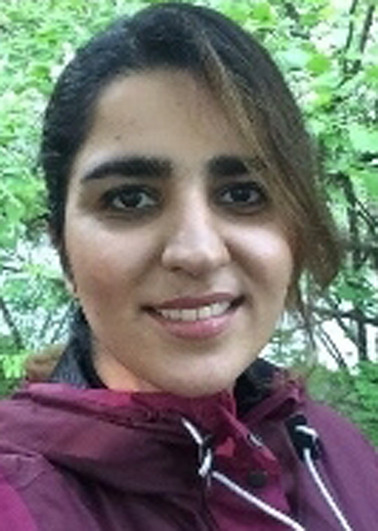

## Biographical Information

*Shima Tavakoli is currently a PhD student in the Department of Polymer Chemistry, Ångström Laboratory, Uppsala University (Sweden) under the supervision of Prof. Oommen Varghese and Prof. Jöns Hilborn. She is early‐stage researcher in the CARTHAGO Marie Curie program, and her project involves the development of crosslinking chemistry to prepare extracellular matrix mimetic hydrogel materials for cartilage regeneration*.

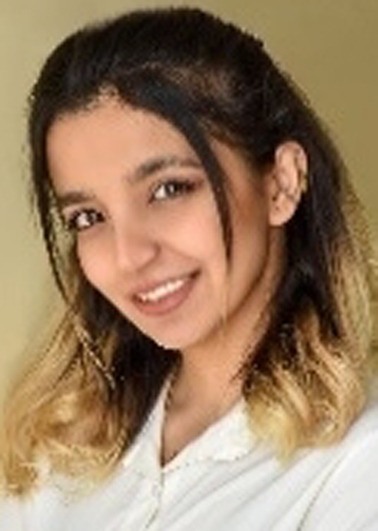

## Biographical Information

*Mahshid Kharaziha received her PhD degree in the Department of Materials Engineering, Isfahan University of Technology (Iran) in 2013. She is currently an Associate Professor of Materials Engineering at Isfahan University of Technology. Her scientific interests focus on biomaterials production, microfabrication processes, and implantable and degradable devices and constructs for biomedical applications*.

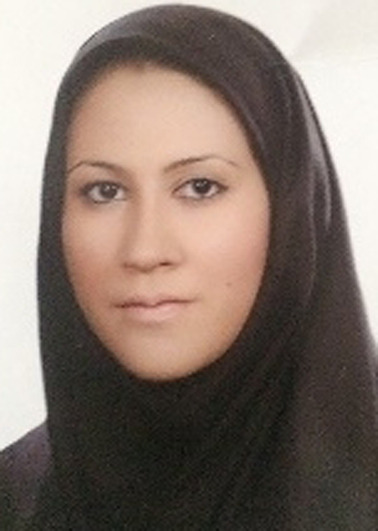

## Biographical Information

*Hubert Girault is Professor of Chemistry at the Ecole Polytechnique Federale de Lausanne (Switzerland). His research activities range from physical electrochemistry for the study of polarised interfaces to bioelectroanalytical chemistry with a focus on bacteria. He is also developing novel redox flow batteries and hydrogen production processes*.

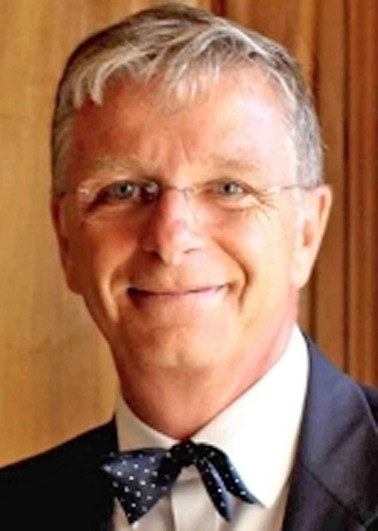

## Biographical Information

*Clemens F. Kaminski FRSC is Professor of Chemical Physics and Head of the Department of Chemical Engineering and Biotechnology at the University of Cambridge (UK). He completed doctoral work at the University of Oxford (Prof. P. Ewart) before he took up a postdoctoral position at the University of Lund (with Prof. M. Aldén). His research interests centre on the development and application of high‐resolution optical imaging methods to elucidate molecular mechanisms of disease*.

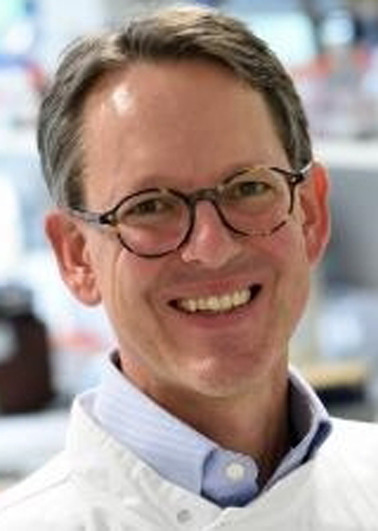

## Biographical Information

*Ioanna Mela received her PhD in Pharmacology at the University of Cambridge (UK) and is currently a Research Associate in the Department of Chemical Engineering and Biotechnology at the University of Cambridge (Prof C. F. Kaminski). Her research interests focus on the design of DNA nanostructures for targeted antimicrobial delivery and on the development of correlative atomic force with super‐resolution microscopy platforms*.